# Structural, biochemical and bioinformatic analyses of nonribosomal peptide synthetase adenylation domains

**DOI:** 10.1039/d3np00064h

**Published:** 2024-03-15

**Authors:** Stephanie C. Heard, Jaclyn M. Winter

**Affiliations:** a Department of Pharmacology and Toxicology, University of Utah Salt Lake City UT 84112 USA sheard@uic.edu Jaclyn.winter@utah.edu

## Abstract

Covering: 1997 to July 2023

The adenylation reaction has been a subject of scientific intrigue since it was first recognized as essential to many biological processes, including the homeostasis and pathogenicity of some bacteria and the activation of amino acids for protein synthesis in mammals. Several foundational studies on adenylation (A) domains have facilitated an improved understanding of their molecular structures and biochemical properties, in particular work on nonribosomal peptide synthetases (NRPSs). In NRPS pathways, A domains activate their respective acyl substrates for incorporation into a growing peptidyl chain, and many nonribosomal peptides are bioactive. From a natural product drug discovery perspective, improving existing bioinformatics platforms to predict unique NRPS products more accurately from genomic data is desirable. Here, we summarize characterization efforts of A domains primarily from NRPS pathways from July 1997 up to July 2023, covering protein structure elucidation, *in vitro* assay development, and *in silico* tools for improved predictions.

## Abstract 2

This review highlights the utility of using adenylation domain structural data, biochemical assays, and computational predictions for prioritizing nonribosomal peptide pathways for natural product discovery.

## Graphical abstract

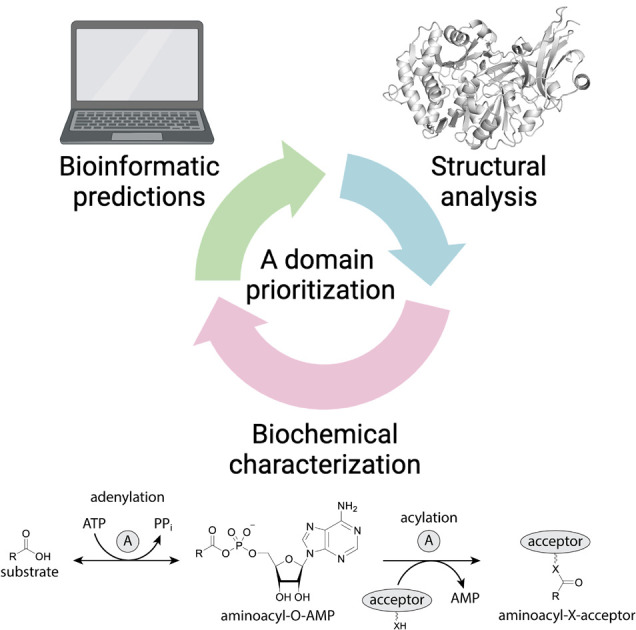

## 1 Introduction

**Fig. 1:**
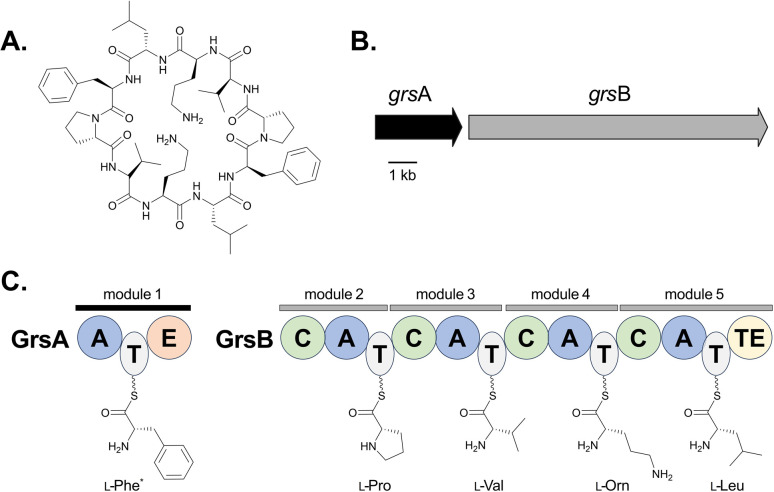
Overview of gramicidin S biosynthesis. (A) Structure of gramicidin S. (B) NRPS-encoding genes from the *grs* cluster found in the genome of *Brevibacillus brevis*.^177–179^ (C) Domain organization of the core enzymes GrsA and GrsB, which use l-amino acids to build gramicidin S.^179^ A = Adenylation, T = thiolation, C = condensation, E = epimerization, TE = thioesterase. *The loading of l-Phe by module 1 is drawn, and it should be noted that the E domain catalyzes epimerization of this residue to d-Phe before it is transferred to l-Pro.

Adenylation is a common and essential reaction across biology involving the covalent attachment of a molecule of adenosine monophosphate (AMP) to a carboxyl-containing substrate. Adenylate-forming enzymes can be classified into three groups: class I comprises luciferases, aryl- and acyl-CoA synthetases, and fatty acid-AMP ligases, while class II includes aminoacyl-tRNA synthetases required to generate charged tRNAs for protein translation *via* the ribosome, and class III contains adenylating enzymes found in nonribosomal peptide synthetase-independent siderophore (NIS) pathways.^1–4^ Adenylation domains found in natural product biosynthetic pathways, specifically those associated with nonribosomal peptide synthetases (NRPSs), are part of class I and will be the focus of this review. Unlike ribosomal-derived peptide synthesis, NRPSs use a thio-templated assembly line-like approach to generate their products.^5,6^ Based on the organization of the catalytic domains, NRPSs can be delineated as either type I or type II, with type I enzymes being large modular megasynthetases encoding multiple catalytic domains that typically function in a colinear manner. In type I NRPS systems, each module is responsible for the activation, incorporation and modification of a single building block, and modules can be further broken down into domains, each containing an independent active site with a defined function in the biosynthesis of a peptide product (Fig. 1).^7^

In type I NRPS systems, a minimal chain elongation module contains adenylation (A), thiolation (T) and condensation (C) domains. The primary gatekeepers to substrate incorporation are the A domains,^8^ as they are responsible for selectively activating building blocks and tethering them to a neighboring T domain in a two-step reaction. T domains, or peptidyl carrier proteins (PCPs), are converted to their holo form by addition of a flexible phosphopantetheine (Ppant) arm. This arm facilitates the delivery of the building block to the C domain, where it is coupled with the upstream nascent peptide. Formation of the amide bond is catalyzed through a Claisen condensation reaction and it has been posited that C domains act as secondary gatekeepers in the generation of nonribosomal peptides (Fig. 1).^8–12^ Additional domains found within a module, such as epimerization (E), methyltransferases (MT), and oxidation (Ox) domains, can further modify a building block. Following assembly of the NRP, the product can be offloaded from the megasynthetase by one of several mechanisms: modified C domains can catalyze cyclization (Cy) of the linear peptide into a macrolactam or macrolactone, reductive (R) domains can release the free aldehyde, or thioesterase (TE) domains can either hydrolyze the chain as a linear peptide or facilitate macrocyclization.^6,7^ To enable customized peptide synthesis, extensive efforts have been dedicated to the bioengineering of modules within type I NRPS assembly lines.^13–15^

**Fig. 2:**
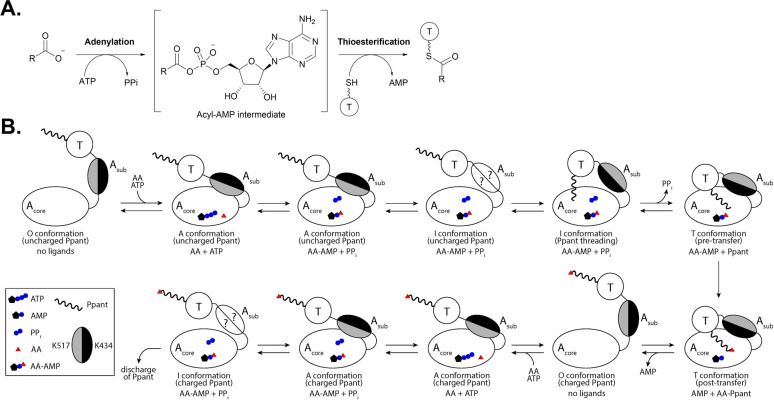
The catalytic cycle of an adenylation reaction. (A) An overview of the general two-step adenylation/thioesterification reaction. (B) The conformational changes that occur between the A_core_ and A_sub_ regions of an adenylation domain upon interaction with its substrates and a neighboring T domain. Adapted from ref. 26 and 27. ATP = Adenosine triphosphate, AMP = adenosine monophosphate, PP_i_ = inorganic phosphate, AA = acyl substrate, AA-AMP = acyl-adenosine monophosphate intermediate, Ppant = phosphopantetheine, O = open, A = adenylation, I = intermediate, and T = thiolation.

For the selection and activation of building blocks, the adenylation reaction proceeds in two steps. First, adenosine monophosphate (AMP) is transferred to the substrate *via* a nucleophilic attack of the carboxylate on the α-phosphate group of adenosine triphosphate (ATP), releasing inorganic pyrophosphate (PP_i_) and generating a reactive acyl-adenylate intermediate (Fig. 2A). The acyl-AMP intermediate is then transferred to a nucleophilic acceptor containing either a thiol, alcohol or amine group, releasing AMP and generating the corresponding thioester, ester or amide, respectively.^1,2^ In NRPS assembly lines, the activated acyl-AMP intermediate is then loaded onto the Ppant prosthetic group of a neighboring T domain (Fig. 2A), from where it can be added to the growing peptide chain by the C domain.^6,7,16^ The complex conformational changes required for successful substrate incorporation in an NRPS assembly line include the concerted participation of A, T and C domains for the complete catalytic cycle (Fig. 2B) and were illuminated when the first structure of an intact NRPS module, SrfA-C, was solved.^17^

**Fig. 3:**
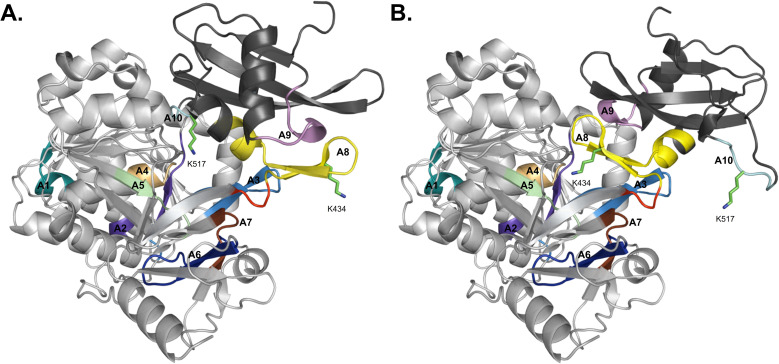
Domain alternation of the A_core_ and A_sub_ regions observed in AlphaFold2 models of GrsA_A. (A) The closed/adenylation conformation and (B) open/thiolation conformation. The A_core_ subdomain is colored light gray whereas the A_sub_ subdomain is colored dark gray. Motifs A1–A7 are found in A_core_, while A_sub_ contains motifs A8–A10. The motifs are colored coded as A1: teal, A2: purple, A3: sky blue, A4: sand, A5: pale green, A6: dark blue, A7: dark brown, A8: yellow, A9: violet, and A10: aquamarine. Lysine residues 517 and 434 are shown in stick form and the hinge region is highlighted in red.

Structurally, adenylation domains can be divided into two lobes, a large N-terminal region (A_core_) and a smaller C-terminal portion (A_sub_). The ten core motifs of adenylating enzymes are divided between these subdomains, with motifs A1–A7 being found in A_core_ and A_sub_ containing motifs A8–A10 (Fig. 3).^5,18^ A hinge region (GRxD) at the beginning of motif A8 separates the two subdomains and has the ability to undergo rigid-body rotation, thereby allowing the A domain to adopt several conformations. After substrate binding, a closed conformation facilitates adenylation and retains the highly reactive intermediate until it can be transferred to the Ppant arm on an adjacent thiolation domain. Switching to the thiolation conformation requires A_sub_ to rotate about 140° at the hinge region, assuming a more open conformation for the second half reaction to proceed. This process is known as the domain alternation hypothesis,^4,19,20^ and the 140° rotation can be seen in Fig. 3, where motifs A1–A7 in A_core_ are almost identical between the two conformations, in contrast to A8–A10 of A_sub_. Notably, two key lysine residues that stabilize the adenylation and thiolation half reactions (K517 in A10 and K434 in A8, respectively, in the GrsA_A models) become near superimposable with their counterparts (K517 of the A conformation with K434 of the T conformation, and K434 of the A conformation with K517 of the T conformation) upon alignment of both structures. The rotation of A_sub_ into the thiolation confirmation is believed to facilitate the escape of PP_i_ from the active site. Subsequently, the vacated space is filled by a conserved salt bridge (between R439 in A8 and E327 in A5), serving as a control switch.^20,21^ When an A domain is in the T conformation, it creates a new surface for protein–protein interaction with the recruited T domain. There has been significant work supporting domain alternation as the mechanism governing A domain conformational dynamics, especially as part of an intact module.^22–29^

Though there does appear to be a spectrum of promiscuity that A domains exhibit, they are generally accepted as the primary gatekeepers to NRPS biosynthetic pathways and contribute to the initial structural diversity of nonribosomal peptides.^8^ Despite a lack of structural similarity, A domains from NRPSs are mechanistically similar to fatty acid synthases^30^ and aminoacyl-tRNA synthetases,^31^ in that they are responsible for the selection and activation of extender units in biosynthetic assembly lines.^1,32^ Remarkably, despite the large pool of known monomers that NRPSs can incorporate (estimated to be >300),^33^ many A domains have high substrate selectivity, and extensive work has sought to define the structural features of the binding pocket that allows for this level of molecular discrimination.^18,34–39^ From the earliest reports of A domain crystal structures, a “code” of minimally required residues was identified in the active site and ascribed to either key contacts with conserved atoms at the N- and C-termini or substrate discrimination with various side chain chemistries. These selectivity-conferring residues have since been incorporated into several predictive algorithms and web tools for the improved annotation of NRPS gene clusters. Naturally, *in silico* predictions must be supported by empirical biochemical evidence to improve computational methods and augment existing knowledge.

This review summarizes the efforts to characterize adenylation domains from natural product NRPS pathways, with a few notable exceptions. The topics covered include the analysis of structural data and determination of substrate selectivity “codes,” the variety of *in vitro* methods available for the biochemical characterization of A domains, and a comparison of available *in silico* tools for predicting A domain substrate selectivity in NRPS pathways, which has become standard practice in the post-genomic era. We will not be discussing NRPS pathway engineering strategies, which have been extensively reviewed elsewhere.^13–15,40,41^ Regarding the timing of substrate modifications, all A domains discussed herein load substrates that have been biosynthesized and/or modified prior to incorporation into the assembly line. We will not be addressing modifications that are installed by the NRPS during peptide extension (*i.e.* epimerization, cyclization, methylation, *etc.*), which are dictated by the embedded domain architecture, or post-assembly line tailoring reactions (*i.e.* methylation, halogenation, oxidoreduction, *etc.*), which are installed by discrete enzymes found either nearby in the gene cluster or elsewhere in the genome. The timing and enzymatic origin of these modifications in a final product can generally be discerned by careful annotation and thorough biochemical characterization of the NRPS and its gene cluster. This review covers from July 1997 up to July 2023.

## 2 Structural analysis

**Fig. 4:**
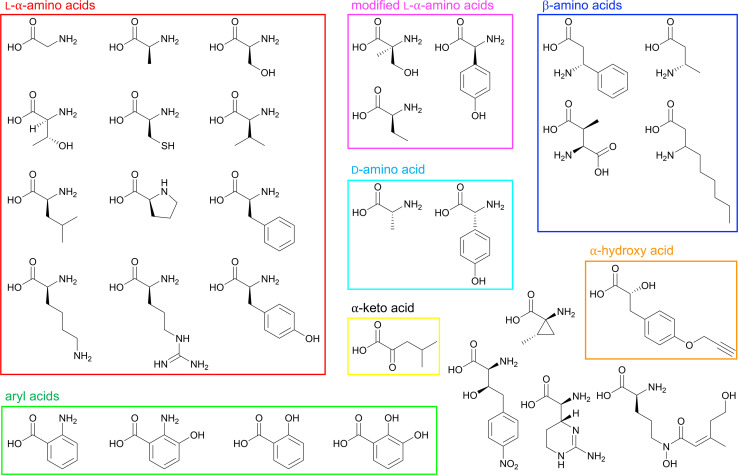
Structures of various proteinogenic and nonproteinogenic building blocks identified in the NRPS pathways whose structures are listed in Table 1. Boxed in red are l-α-amino acids, modified l-α-amino acids are boxed in pink, the d-amino acid is in cyan, β-amino acids are boxed in blue, aryl acids are shown in green, and the α-hydroxy acid and α-keto acid are boxed in orange and yellow, respectively. Structures not in boxes do not belong to any specific group.

A deeper understanding of molecular interactions in binding sites can help guide the rational design of inhibitors or custom-building blocks which, if tolerated and processed by the rest of the assembly line, can help generate new bioactive peptides. The first crystal structure of an NRPS adenylation domain was solved in 1997, and the impact of the detailed analysis of PheA is still recognized to this day.^18,42^ We have chosen to highlight all NRPS structures deposited into the Protein Data Bank (PDB) that contain an A domain (Table 1), many of which are multi-domain proteins or entire modules, and a small subset of these structures have been summarized previously.^43–49^ These protein structures have provided strong evidence for the activation of a wide variety of building blocks (Fig. 4), including putative residues involved in direct substrate interactions or else contributing to the size, shape and chemistry of the binding pocket. Adenylation domains from carboxylate reductase enzymes with the domain organization A-T-R, where R is an NAD(P)H-dependent reductive domain, are beyond the scope of the review and are not discussed as we want to emphasize NRPS pathways.

**Table 1:** All protein structures included in this review, organized by substrate. Adapted from ref. 43 and 49a

Protein	PDB ID	Substrate	Ligands	Final NP	Organism	Year	Notes, ref.
*AlmE	4OXI	Gly	Gly-AMP	LPS modification	*Vibrio cholerae* O1 biovar. El Tor str. N16961	2014	61
AB3404	4ZXH, **4ZXI**	Gly	Gly, AMP	Tyrocidine	*Acinetobacter baumannii* AB307-0294	2016	C-A-T-TE full module^66^
*DhbF (A_1_PCP_1_C_2_)	5U89	Gly	Gly-AVS	Bacillibactin	*Geobacillus* sp. Y4.1MC1	2017	Cross-module with MbtH-like protein^62^
*IdnL7	6AKD	Gly, l-α-Ala, l-α-Ser	Ala-AMS	Incednine	*Streptomyces* sp. ML694-90F3	2019	Prefers small l-AAs^63^
*EntF	5T3D	l-α-Ser	Ser-AVS	Enterobactin	*Escherichia coli* K-12	2016	C-A-T-TE full module with MbtH-like protein^66^
EntF	**5JA1, 5JA2**	l-α-Ser	Ser-AVS	Enterobactin	*Escherichia coli* K-12	2016	C-A-T-TE full module with 2 MbtH-like proteins^67^
Txo1	6OYF, **6OZV**, 6P3I, **6P4U**	l-α-Ser	AMP; Mg^2+^, AMP	Teixobactin	*Eleftheria terrae*	2019	C_2_-A_3_ truncated N-terminal didomain^180^
Txo2	6P1J	l-α-Ser	None	Teixobactin	*Eleftheria terrae*	2019	C_1_-A_1_ didomain^180^
FscH_A	*6EA3*	l-α-Ser	Ser-AMP	Fuscachelins	*Thermobifida fusca* YX	2019	With MbtH-like FscK^181^
*FmoA3	6LTA, **6LTB, 6LTC, 6LTD**	α-me-l-Ser	ANP; α-me-l-Ser-AMP; α-me-l-Ser-AMP	JBIR-34, JBIR-35	*Streptomyces* sp. Sp080513GE-23	2021	Cy-A-T full module, S1046A mutant^68^
*Thr1	5N9W, **5N9X**	l-α-Thr	l-Thr, ATP, Thr-AMP	4-Chloro-Thr	*Streptomyces* sp. OH-5093	2017	65
*PchE	**7EMY, 7EN1, 7EN2**	l-α-Cys	Sal, Cys-AMP; J9F, Sal, Cys-AMP; AMP	Pyochelin	*Pseudomonas aeruginosa* PAO1	2021	T-Cy-A-E-T interrupted elongation module by cryo-EM^69^
BmdB	7LY4, **7LY7**	l-α-Cys	Cys-AVS	Bacillamides	*Thermoactinomyces vulgaris*	2022	Cy_2_-A_2_-T_2_ complex with BmdC, cryo-EM and X-ray^182^
BmdB_A2	7LY5	l-α-Cys	None	Bacillamides	*Thermoactinomyces vulgaris*	2022	Complex with BmdC^182^
PA1221	4DG8, **4DG9**	l-α-Val	Val-AVS	Unknown	*Pseudomonas aeruginosa* PAO1	2012	A-T didomain^183^
LgrA	5ES5, 5ES6, **5ES7, 5ES8**, 5ES9	l-α-Val	l-Val, AMPcPP, 5-fTHF; Val-NH-Ppant	Linear gramicidin	*Brevibacillus parabrevis*	2016	F-A-T initiation module^184^
LgrA	5JNF	l-α-Val	None	Linear gramicidin	*Brevibacillus parabrevis*	2016	F-A-T initiation module^185^
TioS (A_4a_M_4_A_4b_)	5WMM	Norcoronamic acid, l-α-Val	Val-AMP, SAH	Thiocoraline	*Micromonospora* sp. ML1	2018	MT domain insertion^186^
LgrA	**6MFW, 6MFX**, 6MFY, 6MFZ, **6MG0**	l-α-Val and Gly	l-Val, fVal-NH-Ppant, AMPcPP, 5-fTHF; l-Val, AMPcPP, fVal-NH-Ppant; Val-AVS	Linear gramicidin	*Brevibacillus parabrevis*	2019	Dimodular F-A-T-C-A-T^187^
LgrA	6ULZ	l-α-Val	α-Kiv, AMPcPP	Linear gramicidin	*Brevibacillus parabrevis*	2020	P483M mutant^84^
SrfA-C	2VSQ	l-α-Leu	None	Surfactin	*Bacillus subtilis*	2008	C-A-T-TE full module^17^
*Tcp9_A1	8GKM	l-α-Leu	l-Leu	Vancomycin-type glycopeptide	*Actinoplanes teichomyceticus*	2023	H237Y/L287M/L295M ancestral mutant^99^
Tcp9_A1	8GJP	l/d-α-Leu	None	Vancomycin-type glycopeptide	*Actinoplanes teichomyceticus*	2023	H237Y/L295V ancestral mutant^99^
PltF	6O6E	l-Pro	Pro-AVS	Pyoluteorin	*Pseudomonas protegens* Pf-5	2020	With PltL T domain^188^
PigI	Unknown	l-Pro	Unknown	Prodigiosin	*Serratia marcescens*	Unreleased	Crosslinked to PigG T domain^189^
*GrsA_A (PheA)	1AMU	l-α-Phe	l-Phe, AMP	Gramicidin S	*Brevibacillus brevis*	1997	42
*McyG	4R0M	l-α-Phe	l-Phe-AMP	Microcystin	*Microcystis aeruginosa* PCC 7806	2015	A-T didomain^60^
*Pls_A	7WEW	l-α-Lys	Lys-AMP	ε-Poly-l-lysine	*Streptomyces albulus*	2022	70
*ApnA_A1	**4D4G**, 4D4H, **4D4I, 4D56, 4D57**	l-α-Arg/Tyr	ANP; l-Arg, ANP; l-Tyr-AMP; l-Arg-AMP	Anabaenopeptin	*Planktothrix agardhii*	2015	Bi-specific domain^71^
*DltA	3DHV	d-Ala	d-Ala-AMP	d-Alanylation of lipoteichoic acid	*Bacillus cereus* ATCC 14579	2008	Carrier protein ligase^73^
DltA	**3E7W, 3E7X**	d-Ala	AMP	d-Alanylation of lipoteichoic acid	*Bacillus subtilis*	2008	Carrier protein ligase^20^
DltA	**3FCC, 3FCE**	d-Ala	Mg^2+^, ATP; ATP	d-Alanylation of lipoteichoic acid	*Bacillus cereus* ATCC 14579	2009	Carrier protein ligase^74^
DltA	4PZP	d-Ala	None	d-Alanylation of lipoteichoic acid	*Bacillus cereus* ATCC 14579	2014	Carrier protein ligase^25^
*DltA	7VHV	d-Ala	ATP	d-Alanylation of lipoteichoic acid	*Staphylococcus aureus* Mu50	2022	Carrier protein ligase^75^
DltA	7R27	d-Ala	d-Ala-AMP	d-Alanylation of lipoteichoic acid	*Lactiplantibacillus plantarum* NC8	2022	Carrier protein ligase^76^
*ANC4_A1	8GLC	d-Ala	None	Pekiskomycin-type glycopeptide	*Actinoplanes teichomyceticus*	2023	Ancestral enzyme^99^
Engineered TycA	**5N81, 5N82**	(*S*)-β-Phe	*O*-propargyl-β-Tyr-AMS; β-phe-AMS	N/A (tyrocidine)	*Brevibacillus parabrevis*	2018	A-T didomain with 5 mutations^80^
HitB-HitD	6M01	(*S*)-β-Phe	Acetyl-*N*-Ppant, ADP	Hitachimycins	*Embleya scabrispora*	2020	A-T didomain^190^
*HitB	**7DQ5, 7DQ6**	(*S*)-β-Phe	β-Phe-AMS; 3-Br-β-Phe-AMS	Hitachimycins	*Embleya scabrispora*	2021	79
SlgN1	4GR4, **4GR5**	(2*S*,3*S*)-β-me-l-Asp	AMPcPP	Streptolydigin	*Streptomyces lydicus*	2013	With MbtH-like domain^191^
*VinN	3WV4, **3WV5, 3WVN**	(2*S*,3*S*)-β-me-l-Asp	β-me-l-Asp; l-Asp	Vicenistatin	*Streptomyces halstedii*	2014	77
*IdnL1	5JJQ	(3*S*)-3-Aba	3-Aba-AMP	Incednine	*Streptomyces* sp. ML694-90F3	2017	78
CmiS6	5JJP	3-Ana	None	Cremimycin	*Streptomyces* sp. MJ635-86F5	2017	78
CytC1	*3VNQ, 3VNR, 3VNS*	2-Aba	2-Aba, AMP; l-Val, AMP; ATP	Cytotrienin	*Streptomyces* sp. RK95-74	2007	l-Val best substrate^192^
*Tcp9_A1	8GJ4, **8GIC**	d-4-Hpg	l-4-Hpg	Teicoplanin	*Actinoplanes teichomyceticus*	2023	Used for grafting of ancestral residues^99^
*SidN_A3	3ITE	*cis*-AMHO	None	Fungal ferrichromes	*Epichloe festucae* var. lolii	2010	Fungal A domain^38^
*ObiF1	6N8E	(2*S*,3*R*)-hnhF	hnhF	Obafluorin	*Burkholderia diffusa*	2019	C-A-T-TE full module MbtH-like protein^98^
CmnG_A	7XBS, **7XBT, 7XBU, 7XBV**	l-Cap	AMP; l-Cap; AMPcPP	Capreomycin	*Streptomyces mutabilis* subsp. *capreolus*	Unreleased	193
*Engineered TycA_A	7YWJ, **7YWK**	pPLA	AMP	N/A (tyrocidine)	*Brevibacillus parabrevis*	2022	WT and L313P mutant^81^
*StsA_A	**6ULX, 6ULY**	α-Kic	α-Kic-AMP	Cereulide	*Bacillus stratosphericus* LAMA 585	2020	84
StsA	6ULW	α-Kic	None	Cereulide	*Bacillus stratosphericus* LAMA 585	2020	A-KR-T initiation module^84^
*AuaEII	4WV3	Ant	Ant-AMP	Aurachin	*Stigmatella aurantiaca*	2016	CoA ligase^97^
*PqsA	**5OE3, 5OE4, 5OE5, 5OE6**	Ant	Ant-AMP; Ant-AMP; Ant-AMP; 6-fluoro-Ant-AMP	Pseudomonas quinolone signal	*Pseudomonas aeruginosa* PAO1	2017	CoA-ligase, 3 crystal forms of Ant-AMP^50^
*NpsA	6VHT, 6VHU, **6VHV, 6VHW, 6VHX, 6VHZ**	3-Hydroxy-Ant	3-Hydroxy-Ant-N-AMS; 3-HB-N-AMS; 3-hydroxy-Ant-N-AMS; Ant-N-AMS	Tilimycin	*Klebsiella oxytoca*	2020	Full A or truncated N-terminal domain^51^
NpsA	6VHY	3-Hydroxy-Ant	3-HB-N-AVS	Tilimycin	*Klebsiella oxytoca*	2020	A-T didomain fusion with ThdA^51^
*CahJ	**5WM2, 5WM3, 5WM4, 5WM5, 5WM6, 5WM7**	Sal	Sal, AMP; Sal-AMP; 6-me-Sal-AMP; 5-me-Sal-AMP; benzoate-AMP; AMP	Cahuitamycins	*Streptomyces gandocaensis*	2018	96
PchD	**7TYB, 7TZ4**	Sal	Sal-AMS; 4-cyano-Sal-AMS	Pyochelin	*Pseudomonas aeruginosa* PAO1	2022	194
MbtA	5KEI	Sal	None	Mycobactin	*Mycobacterium smegmatis*	2016	195
*DhbE	**1MD9, 1MDB**, 1MDF	2,3-Dhb	2,3-Dhb, AMP; 2,3-Dhb-AMP	Bacillibactin	*Bacillus subtilis*	2002	87
BasE	**3O82, 3O83, 3O84**	2,3-Dhb	2,3-Dhb-AMS; 2-HB-DTAMS; HTJ	Acinetobactin	*Acinetobacter baumannii* AB900	2010	196
*BasE	**3U16, 3U17**	2,3-Dhb	H89, H90	Acinetobactin	*Acinetobacter baumannii* AB900	2012	Bi-substrate inhibitors^91^
EntE-B	3RG2	2,3-Dhb	Sal-AVS	Enterobactin	*Escherichia coli*	2012	A-T fusion^92^
EntE-B	4IZ6	2,3-Dhb	2,3-Dhb-AVS	Enterobactin	*Escherichia coli*	2013	A-T fusion^93^
*EntE	**6IYK, 6IYL**	2,3-Dhb	2-Nitro benzoyl-AMS; 3-cyano benzoyl-AMS	Enterobactin	*Escherichia coli*	2019	N235G mutant^94^
FscC	*6E97, 6E8O*	2,3-Dhb	2,3-Dhb-AMP; AMP	Fuscachelins	*Thermobifida fusca* YX	2019	181

a Bolded PDB IDs indicate structures from multi-structure reports that are bound to the ligand(s) listed. PDB IDs in italics indicate that no publication has yet to be associated with the structure. Entries marked with “*” are discussed in the text in more detail for contributing to our understanding of substrate binding at the molecular level. PDB = Protein Data Bank, NP = natural product, AA = amino acid, A = adenylation, T = thiolation, C = condensation, Cy = cyclization, TE = thioesterase, E = epimerization, F = formylation, KR = ketoreductase, ATP = adenosine triphosphate, ADP = adenosine diphosphate, AMP = adenosine monophosphate, Ppant = phosphopantetheine, ANP = phosphoaminophosphonic acid-adenylate ester, AMPcPP = diphosphomethylphosphonic acid adenosyl ester, AVS = 5′-(vinylsulfonylamino) adenosine (AMP analog), AMS = 5′-*O*-sulfamoyl adenosine (AMP analog), DTAMS = 2-(4-*n*-dodecyl-1,2,3-triazol-1-yl)-5′-*O*-sulfamoyl adenosine, 5-fTHF = 5-formyltetrahydrofolate, f = formyl, me = methyl, Aba = aminobutyric acid, Ana = aminononanoic acid, Ant = anthranilic acid, Cap = capreomycidine, *cis*-AMHO = *N*^δ^-*cis*-anhydromevalonyl-*N*^δ^-hydroxy-l-ornithine, Dhb = dihydroxybenzoic acid, HB = hydroxybenzene, Hpg = hydroxyphenylglycine, HSC = *N*^1^-hydroxy-*N*^1^-succinyl-cadaverine, α-Kic = α-ketoisocaproic acid, Kiv = ketoisovaleric acid, LPS = lipopolysaccharide, pPLA = 4-propargyloxy-(*S*)-3-phenyllactic acid, SAH = *S*-adenosyl-l-homocysteine, Sal = salicylate, hnhF = β-hydroxy-*para*-nitro homophenylalanine, HTJ = 6-phenyl-1-(puridin-4-ylmethyl)-1*H*-pyrazolo[3,4-*b*]pyridine-4-carboxylic acid, H89 = 6-[4-(benzyloxy)phenyl]-1-(pyridine-4-ylmethyl)-1*H*-pyrazolo[3,4-*b*]pyridine-4-carboxylic acid, H90 = 6-(4-benzoylphenyl)-1-(pyridine-4-ylmethyl)-1*H*-pyrazolo[3,4-*b*]pyridine-4-carboxylic acid, J9F = (4*S*)-2-(2-hydroxyphenyl)-4,5-dihydro-1,3-thiazole-4-carboxylic acid.

Adenylation domains by themselves have been historically limited to X-ray crystallography due to their size (∼50 kDa), which makes them too big for solution-phase nuclear magnetic resonance (NMR) spectroscopy and too small for current cryo-electron microscopy (cryo-EM) techniques. Some A domain crystal structures have only been obtained by removing the C-terminal A_sub_ region completely (PqsA, PDB 5OE3; and NpsA, PDB 6VHW, 6VHX, and 6VHZ),^50,51^ by trapping the A domain in one conformation through the use of synthetic affinity probes,^52^ or using a combination of these approaches. Before a more comprehensive understanding of the conformational dynamics of A domains, many crystal structures depicted A domains in various poses that were not always easily reconciled. However, with the recent growing interest in studying domain–domain interactions and conformational flexibility of intact NRPS modules, cryo-EM has become an essential technique. Despite the majority of existing structures being generated through X-ray crystallographic studies, the field as a whole seems to be moving towards understanding the complicated dynamics of NRPS megasynthetases.^49,53,54^

**Fig. 5:**
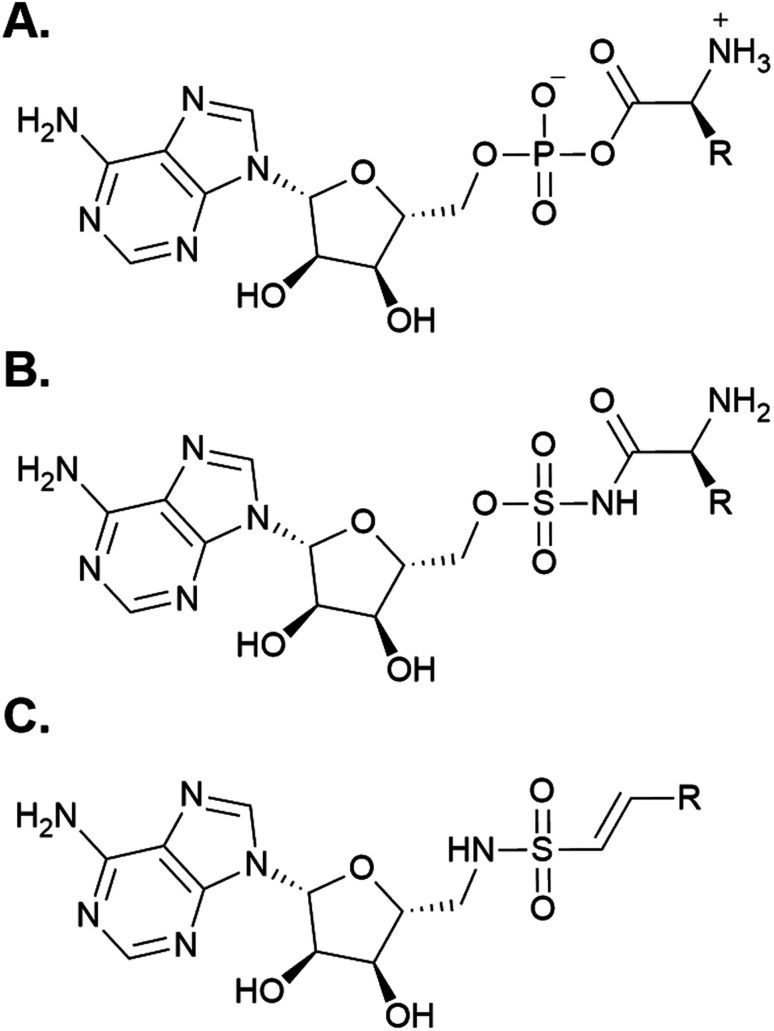
Chemical mimics of AMP used for structure determination of A domains. (A) The structure of AMP. (B) A generic 5′-*O*-sulfamoyl adenosine (AMS) probe. (C) A generic 5′-(vinylsulfonylamino) adenosine (AVS) probe.

Following the development of various inhibitors and affinity probes that trap the protein in the adenylation or thiolation conformations, significant advances have been made in the crystallization of A domain-containing constructs.^52^ Using a bioisosteric analog of AMP (Fig. 5A), 5′-*O*-sulfamoyl adenosines (AMS) were first developed for inhibition of NRPS systems that produced virulence factors (*i.e.* siderophore production in pathogens) (Fig. 5B).^55,56^ AMS inhibitors can be extended with a variety of acyl substrates for the inhibition of any NRPS of interest once its substrate selectivity is known. After the success of AMS inhibitors, dead-end affinity probes were developed using a 5′-(vinylsulfonylamino) adenosine (AVS) moiety (Fig. 5C).^57^ These probes covalently tether to Ppant, trapping A domains in the thiolation/substrate donation conformation. Several variations on both AMS and AVS probes have been subsequently used in crystallography to illuminate the various subtle shifts in protein structure throughout the catalytic cycle.

It should be noted that studies on the conformational changes that occur during the peptide elongation cycle of NRPSs are not limited to structural methods alone. There have been significant contributions from the Mootz group using a FRET-engineered A-T didomain to disentangle the various subdomain movements that occur upon substrate binding, adenylation, and transfer to the phosphopantetheine (Ppant) prosthetic group of the carrier protein.^26,27^ The work describes how an A-T didomain can occupy several different open (O) conformations in the absence of all substrates and at least two distinct closed conformations when substrates are present, with the rotation of A_sub_ delineating the adenylation (A) and thiolation (T) poses.^26^ Between the A and T conformations, there is at least one intermediate (I) conformation that represents the rate-limiting step of aminoacylation, and the full population exists in dynamic equilibrium (Fig. 2B).^27^ Recently, a C-A-T elongation module containing the FRET sensor was generated, and through hydrogen–deuterium exchange mass spectrometry and photolabile steric caging, it was confirmed that multiple enzyme conformations can exist in the bulk reaction mixture, with the binding of some ligands causing a shift in the conformational ratio.^28^ In total, sampling the conformational shifts of multidomain NRPS proteins in solution has revealed that specific substrate binding and transformation events can shift the T domain's affinity for neighboring A and C domains in the direction of templated biosynthesis. Several reviews on the conformational dynamics of multidomain NRPS proteins have been previously published.^4,49,54,58,59^

### 2.1 Activation of l-α-amino acids

Because of their abundance in cells and involvement in many primary metabolic processes, l-α-amino acids are the most common building blocks in NRPS pathways. Consequently, a wealth of l-α-amino acid activating A domain structures have been analyzed, and below we summarize major findings for the different chemical functionalities of some of the 20 proteinogenic amino acids. In general, aliphatic amino acid selectivity is more difficult to determine because of the inherent chemical ambiguity of the residues lining an otherwise nonspecific hydrophobic pocket. Any structurally similar proteinogenic amino acids can face this enigma, with pairings like Gly/Ala, Val/Ile and Ser/Cys exhibiting minor chemical differences that are challenging to differentiate at the enzyme active site level. Some of the reports summarized below list residues that help in atomic-level substrate differentiation, but it remains to be seen if they can be applied more broadly.

**Fig. 6:**
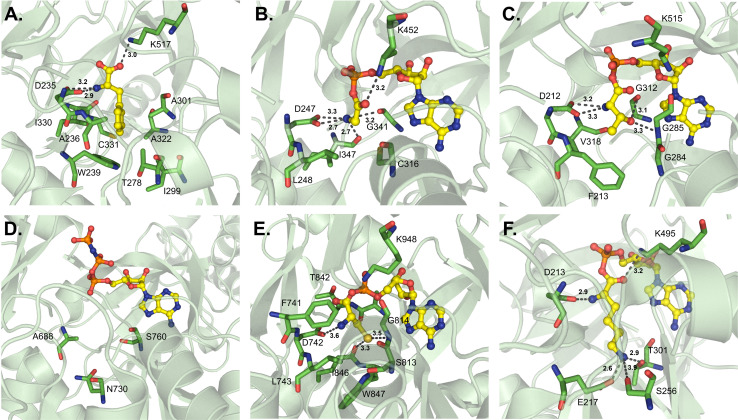
Binding pockets of l-amino acid-activating adenylation domains. Residues involved in substrate interactions are shown for (A) l-α-Phe in GrsA_A (PDB 1AMU); (B) Gly-AMP in AlmE (PDB 4OXI); (C) Thr-AMP in Thr1 (PDB 5N9X); (D) α-me-l-Ser-AMP in FmoA3 (PDB 6LTB); (E) Cys-AMP in PchE (PDB 7EMY); and (F) Lys-AMP in Pls_A (PDB 7WEW). Active site residues are shown as sticks whereas bound substrates are highlighted in yellow. Polar contacts are shown as black dashes.

GrsA_A, also known as PheA, was the first crystallized A domain, identified in the bacterium *Brevibacillus brevis*. The organization of the larger N-terminal A_core_ was reported as having an αβαβα tertiary structure consisting of a distorted β-barrel and two β-sheets interspersed with α-helices. The smaller A_sub_ contains two α-helices and two β-sheets, and both the N- and C-termini are less ordered.^42^ As the first A domain in the gramicidin S biosynthetic pathway, GrsA_A selectively activates and incorporates l-Phe, and its structural report postulated the first set of 10 amino acid residues that lined the binding pocket (the 10AA code). Two of these residues were considered to be invariant and essential for α-amino acid binding and orientation (D235 and K517), but the other eight residues were thought to discriminate between different substrates based on their side chain chemistries (A236, W239, T278, I299, A301, A322, I330 and C331) (Fig. 6A).^42^ The first nine residues of the 10AA code are located in the N-terminal A_core_ region, with only the final K517 being found in the C-terminal A_sub_. Another l-Phe activating A domain, McyG from microcystin biosynthesis, was crystallized in 2015. This study found that V227 (corresponding to D235 in GrsA_A) was essential in the selection of hydrophobic substrates, indicating that an aspartate coordinating the α-amino group is not strictly required, and that residues at the entrance to the pocket can influence substrate discrimination. Further, the benzene ring of Phe was stabilized in the pocket by the side chain of W272 and the backbone atoms of A333, G335 and S341 (McyG numbering).^60^ With the activation of l-Phe, the impact of the identity of the first residue of the 10AA code (Asp *vs.* Val) remains enigmatic. Selectivity benefits for the same substrate may arise from either Asp coordinating the α-amino group or a hydrophobic residue at the entrance of the pocket facilitating side chain interactions.

Several A domain structures have been described that activate glycine or other small l-α-amino acids. AlmE from *Vibrio cholerae* O1 (El Tor biotype) activates Gly to modify lipopolysaccharides (LPS) as a mechanism of resistance to cationic antimicrobial peptides.^61^ Several residues mediate this adenylation reaction: the conserved D247 and K452 coordinate with the glycyl amine and carbonyl groups, respectively (Fig. 6B), and the carbonyl oxygens of G341 and I347 also interact with the amine (AlmE numbering). Specifically, L248 and C316 are close to the C_α_, which might help discourage d-amino acids and/or l-α-amino acids larger than glycine, respectively, from binding through steric interactions. These proposed roles were verified by independent mutagenesis of both L248 and C316 (AlmE numbering).^61^ Tarry *et al.* were able to crystallize a Gly-incorporating A domain as part of a larger cross-module complex. They observed that in DhbF, the conserved D656 (corresponding to D235 of GrsA_A) binds to the amino group of Gly, but otherwise, the substrate binding pocket seemed to be occluded by W755, which also hydrogen bonds with Q699 (DhbF numbering).^62^ A third protein structure that can activate Gly has also been analyzed, but unlike previous examples, IdnL7 shows relaxed substrate selectivity towards small l-α-amino acids (Gly, l-Ala, or l-Ser) with a marked intolerance for d-Ala.^63^ Beyond the conserved first and last positions of its selectivity code (D216 and K500), three additional residues appear to be responsible for this activation pattern: C217, A285 and T318 (IdnL7 numbering). C217 and A285 directly interact with the methyl group of l-Ala, with C217 thought to enforce the stereochemical recognition of l-amino acids over their d-counterparts, while T318 is adjacent to the methyl group and positioned in such a way that accommodation of l-Ser can be rationalized. As it happens, these three residues are highly conserved in VinM-type enzymes.

Incorporation of l-Thr has been described with the free-standing enzyme Thr1 en route to 4-chloro-Thr biosynthesis. Briefly, Thr1 activates l-Thr which is loaded on to the free-standing Thr2 carrier protein, halogenated by Thr3, and offloaded by the thioesterase Thr4.^64^ Upon crystallization, it was noted that D212 and K515 interact with the amine and carbonyl, respectively, and the methyl group of Thr is within van der Waals distance of F213 and G284 (Thr1 numbering) (Fig. 6C). Hydrogen bond interactions are formed between the hydroxyl group and the side chain of H119 and the backbone carbonyls of M310 and V318. But upon adenylation and formation of Thr-AMP, the positioning shifts so that the hydroxyl group is stabilized instead by the backbone amine of G285 and carbonyl of G312 (Thr1 numbering).^65^ An isomer of l-Thr, α-me-l-Ser is activated by the A domain of FmoA3 in the biosynthesis of the radical scavengers JBIR-34 and JBIR-35. It was proposed that residues A688, N730 and S760 of FmoA3 (Fig. 6D), which correlate to V649, H691 and S722 of the l-Ser activating EntF,^66,67^ were important for recognizing the side chain of α-me-l-Ser. These key contacts were verified by mutagenesis experiments, with a particular emphasis on A688 being not only hydrophobic but also small enough to accommodate an α-methyl substituent.^68^ Regarding l-Ser activation, the EntF A domain was crystallized as part of a larger C-A-T-TE module, with only a brief mention of the binding pocket containing D648, S722 and D754.^66^

In pyochelin biosynthesis, PchE activates l-Cys, which is epimerized by the embedded E domain after binding. The predicted specificity code was DLFNLSLIWK, but the identified binding pocket residues were F741, D742, L743, S813, G814, A841, T842, I846, W847 and K948 (PchE numbering) (Fig. 6E). Out of these residues, two pairs of hydrophobic interaction partners (I846/W847 and F741/L743) narrow the pocket to provide Cys-binding specificity,^69^ similar to AB3403.^66^ Because of minor differences in side chain size and polarity, it is not well understood how A domains are able to discriminate between l-Cys and l-Ser. The incorporation of l-Lys into ε-poly-l-lysine is performed by Pls_A. Outside of D213 and K495, which interact with the amine and carbonyl moieties, respectively, E217 locks the ε-amino group at the base of the pocket *via* a salt bridge, along with T301 and S256 *via* hydrogen bonding (Pls_A numbering) (Fig. 6F).^70^ And, from the anabaenopeptin pathway, ApnA_A1 is the only crystallized A domain to date that displays dual specificity towards both l-Arg and l-Tyr. Substrate orientation is maintained by E204 and S243, and A307 (corresponding to C331 in GrsA_A) interacts with the aliphatic chain of Arg and the phenyl ring of Tyr. E204 is conserved in naturally-occurring homologues of ApnA_A1, and the mutation S243H switched the specificity 100-fold to 4-azidophenylalanine (ApnA_A1 numbering).^71^

### 2.2 Activation of d-amino acids

**Fig. 7:**
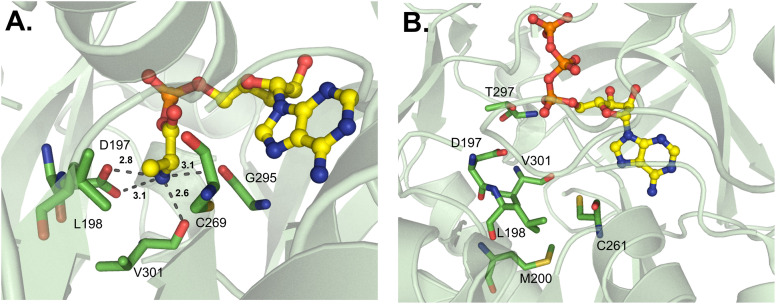
d-Amino acid-activating adenylation domains. (A) Residues interacting with d-Ala-AMP in DltA from *Bacillus cereus* (PDB 3DHV). (B) Residues interacting with ATP in DltA from *Staphylococcus aureus* (PDB 7VHV). Active site residues are shown as sticks whereas bound substrates are highlighted in yellow. Polar contacts are shown as black dashes.

d-Amino acids are just one example of how NRPS assembly lines have evolved for the incorporation of nonproteinogenic amino acids. Thus far, the only d-amino acid activating enzyme that has been crystallized is DltA, which is responsible for the d-alanylation of cell wall components like lipoteichoic acid in Gram-positive bacteria.^72^ This is reminiscent of the glycylation of lipopolysaccharides described above, and moreover, AlmE shows both active site similarity to DltA and relaxed substrate selectivity towards d-Ala.^61^ DltA has been crystallized several times from various organisms, and separate investigations of the active site binding pocket broadly corroborate each other.^20,25,73–76^ The first of these reports identified several residues important for coordination of both the α-amino group (D197, G295 and V301) and the d-methyl group (L198 and C269) in the *Bacillus cereus*-derived enzyme (Fig. 7A).^73^ The most significant of these is C269, which would sterically clash with l-Ala if it were to bind in the active site. Indeed, these residues were almost identical when compared to those of the *Staphylococcus aureus* structure (Fig. 7B), which recognizes the α-amino group with residues D197, T297 and V301 and the methyl side chain with residues L198, M200 and C268 (numbering from the *B. cereus*-derived structure).^75^ In AlmE, two key residues were thought to allow for Gly-specific activation (L248 and C316), with L248 restricting the access of D-substrates (corresponding to L198 in DltA). The conformation of L248 was found to be influenced by interactions with surrounding residues. In DltA, two of these neighboring positions are replaced with smaller side chains (Ser to Ala and Leu to Thr), potentially creating sufficient space for the methyl moiety of d-Ala. Further, the helix of AlmE containing L248 is straight, whereas the corresponding feature in DltA is bent, creating additional space for the methyl side chain. Despite AlmE displaying promiscuity for d-Ala activation, this residue was never successfully transferred to the downstream T domain, and crystals could not be obtained with the replacement of Gly with d-Ala.^61^ Indeed, DltA displays an increased affinity for its native substrate d-Ala, and concomitant decreased affinity for l-Ala, in the presence of coenzyme A (CoA) as a Ppant mimic or its cognate Ppant-modified T domain.^25,75^

### 2.3 Activation of β-amino acids

**Fig. 8:**
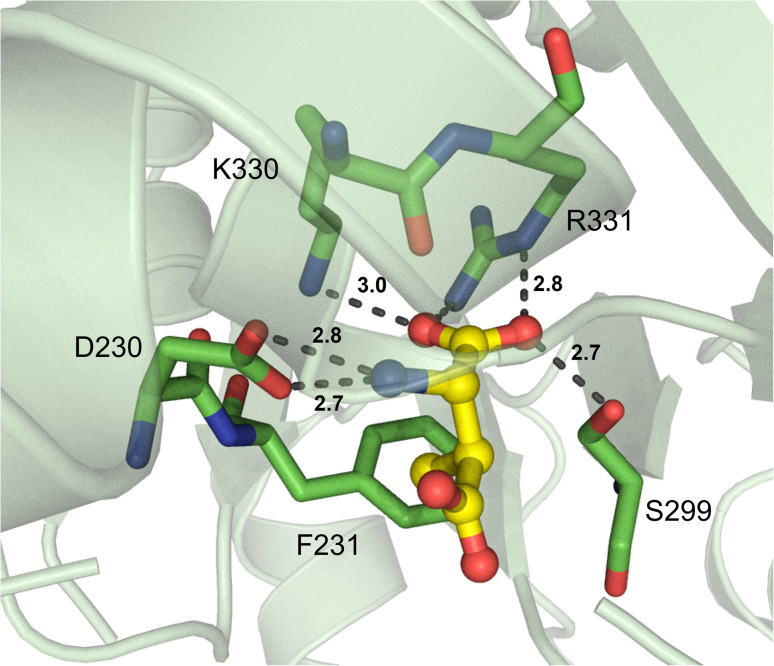
β-Amino acid-activating adenylation domain. Residues interacting with β-me-l-Asp in the active site of VinN are highlighted (PDB 3WV5). Active site residues are shown as sticks whereas β-me-l-Asp is highlighted in yellow. Polar contacts are shown as black dashes.

Another type of nonproteinogenic amino acid that is occasionally used by NRPSs is β-amino acids. The first structure of a β-amino acid activating A domain was observed in VinN, which binds (2*S*,3*S*)-3-methylaspartate (3-MeAsp) as a β-amino acid.^77^ There were two key structural differences that allowed VinN to specifically activate its substrate: the β13β14 loop is one residue shorter than other l-α-amino acid A domains, and two amino acids opposite the loop are replaced by residues with bulkier side chains. The β13β14 loop contains the key residues K330 and R331 (I330 and C331 in GrsA_A), and its shortening shifts the backbone atoms to provide space for the C1–C2 bond in 3-MeAsp. The two residues opposite this loop in VinN are F231 and S299 (Fig. 8), in contrast to A236 and A301 in GrsA_A, and it is thought that these larger residues might push the β-amino acid substrate closer to the β13β14 loop. F231, in particular, was assessed by mutagenesis experiments and confirmed to be important for binding of both 3-MeAsp and l-Asp in VinN. The β-amino group was found to be coordinated by the conserved D230 present in many adenylating enzymes (D235 in GrsA_A).^77^ Despite this report noting that another β-amino acid activating A domain, SlgN1, binds 3-MeAsp in the opposite orientation to VinN, structural characterization of other β-amino acid A domains has supported the findings of VinN. Specifically, IdnL1 and CmiS6 seem to recognize their substrates *via* the same mechanism as VinN.^78^ The only new insight gained from this follow-up report is that in IdnL1 and CmiS6, position 220 (IdnL1 numbering) seems to be important in dictating the size of substrate that they can accommodate. L220 of IdnL1 helps recognize (*S*)-3-aminobutanoic acid ((*S*)-3-Aba), while G220 of CmiS6 expands the binding pocket to accommodate 3-aminononanoic acid (3-Ana).

A study aimed at generating analogs of the β-amino acid-containing hitachimycins investigated the tolerability of different substituents on (*S*)-β-Phe in the adenylation reaction of HitB. HitB contains all the β-amino acid-specific A domain motifs discussed above, and it was found to have reasonable tolerance for a wide range of alternative substrates. Upon solving the structure of HitB in complex with (*S*)-β-3-bromo-Phe-AMS, it was noted that the side chain of F328 was flexible (HitB numbering), allowing for the activation of *meta*-substituted (*S*)-β-Phe analogs.^79^

Additional studies on β-amino acid activating A domains led to the reprogramming of an α-amino acid A domain and swapped its functionality. Niquille and co-workers have used fluorescence activated cell sorting (FACS) to enable rapid screening of a yeast cell surface display library of TycA mutants.^80^ They replaced the four-residue β13β14 loop with a randomized tripeptide, and at the opposite side of the pocket, A236 was randomized to account for other structural mutations (GrsA_A numbering). Of all the variants that were sequenced, the mutation A236V was 100% conserved, and the sequence of the β13β14 loop converged to the motif Xaa-Leu-Val (where Xaa is Ala, Thr, Cys, Val or Leu). Upon structural analysis, it was found that the conserved D235 interacted with the substrate β-amino group, as observed in VinN above. The randomized residues at position 236 and in the β13β14 loop were deemed essential for the α/β-specificity switch, which allowed for the measurement of a 40 000-fold increase in TycA preference for (*S*)-β-Phe over l-Phe.^80^

### 2.4 Activation of α-hydroxy acids

**Fig. 9:**
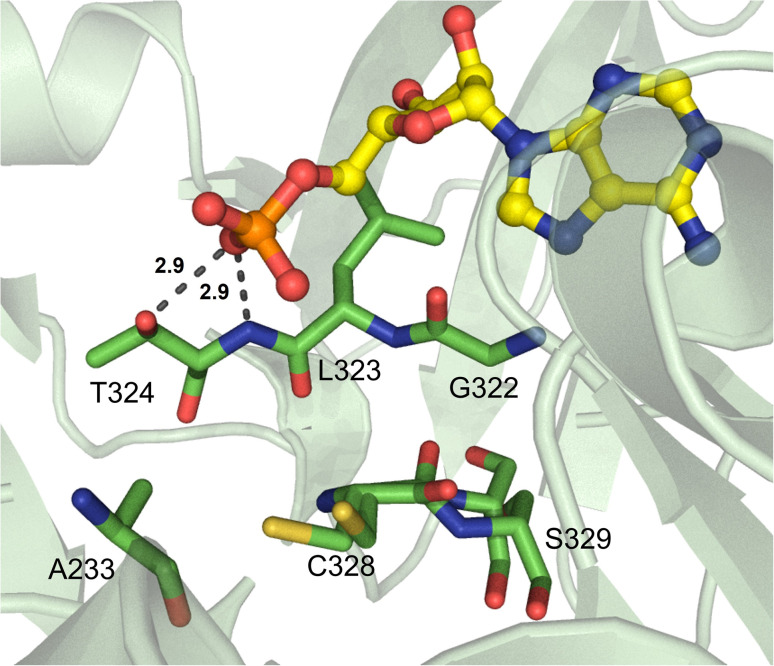
α-Hydroxy acid-activating adenylation domain. Residues interacting with AMP in the active site of the engineered TycA_A variant are highlighted (PDB 7YWK). Active site residues are shown as sticks whereas AMP is highlighted in yellow. Polar contacts are shown as black dashes.

An α-amino group is not absolutely required for A domain substrate activation: in fact, many NRPS enzymes activate monomers containing an α-hydroxy functional group. However, only one α-hydroxy acid A domain has ever been crystallized to date, and it was an engineered variant of TycA from tyrocidine biosynthesis (Fig. 9).^81^ The authors used combinatorial mutagenesis and yeast cell surface display selection for the high-throughput generation of TycA variants with preferential selectivity for phenyllactic acid (PLA) over the native l-Phe substrate. They also exploited a point mutation, W227S, which is known to allow for the incorporation of “clickable” amino acids, specifically by accommodating a 4-proparagyloxy substituent in the binding pocket.^82^ It was found that three additional positions in the binding pocket were critical for recognition of the α-hydroxy substrate, namely P313, C318 and A223 (TycA numbering). A223 would normally be the highly conserved aspartate residue that is involved in the recognition of the α-amino group of an amino acid building block, but its replacement with a small, neutral amino acid allowed for preferential binding of PLA substrates. C318, which replaced an isoleucine, is thought to use its side chain to hydrogen bond to the PLA substrates. P313 is a conformationally strained residue that, when replaced with the native leucine, would provide a backbone NH that is within hydrogen bonding distance to the backbone carbonyl of residue 318, making it unavailable for interaction with the substrate.^81^ The L313P mutation was serendipitous in this study, but it highlighted the significance of the backbone atoms in substrate orientation and binding in the active site pocket.

Though no native α-hydroxy acid-bound A domain structures have yet been described, homology modeling and substrate docking can provide a basis for structure-guided mutagenesis. Hoffman and co-workers generated a structure model of EnSynA_1_, which activates d-α-hydroxyisovaleric acid in enniatin biosynthesis, and biochemical characterization of several mutants revealed the importance of key residues. These broadly confirmed the structural findings described above, with G680 (EnSyn numbering) being required for substrate access at the entrance of the binding cavity much like A223 in engineered TycA, and S773 and G767 (EnSyn numbering) providing critical hydrogen bonds through their backbone carbonyl atoms like L313 in native TycA.^83^ In both of these examples of α-hydroxy acid-activating A domains, the first residue of the 10AA selectivity code (D235 in GrsA_A) is a smaller, aliphatic residue – Ala in TycA and Gly in EnSynA_1_ – similar to the Phe-activating McyG, which maintains Val in this position.

### 2.5 Activation of α-keto acids

**Fig. 10:**
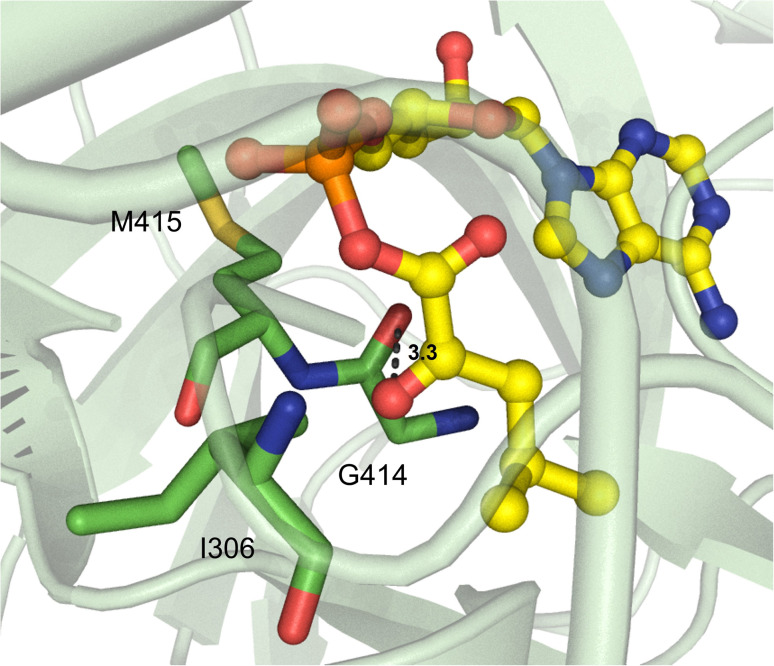
α-Keto acid-activating adenylation domain. Residues interacting with α-ketoisocaproic acid-AMP (α-Kic-AMP) in the active site of StsA_A are highlighted (PDB 6ULX). Active site residues are shown as sticks whereas α-Kic-AMP is highlighted in yellow. Polar contacts are shown as black dashes.

Beyond α-hydroxy acid activation, some NRPS pathways, including depsipeptide synthetases, incorporate α-keto acid building blocks. There is only one example of a crystallized α-keto acid A domain, which is the α-ketoisocaproic acid (α-Kic) selecting StsA_A from cereulide biosynthesis. This A domain structure was obtained as part of a larger study on depsipeptide synthetase modules, but there were some striking substrate interactions that were reported.^84^ Similar to previous reports on α-keto acid A domains,^85,86^ the α-amino contacting aspartate residue in StsA_A has been replaced with the hydrophobic residue I306 (StsA_A numbering), which is reminiscent of the α-hydroxy acid A domains discussed above. From this structure, the only other molecular contact that was deemed essential for α-keto acid binding was the backbone carbonyl between G414 and M415 (Fig. 10). Of note, M415 is a conserved proline in α-amino acid activating A domains, and the M415P mutant abolished adenylation activity of StsA_A. Mutation of this residue in linear gramicidin synthetase A (LgrA) to either Met or Ala (P483M, P483A) led to a small but significant increase in adenylation activity towards α-ketoisovaleric acid (α-Kiv), the α-keto equivalent of its native substrate l-Val.^84^ This work further highlights the importance of the shape and backbone atom orientations in A domain binding pockets.

### 2.6 Activation of aryl acids

There is an abundance of NRPSs that incorporate aryl acids into their final products, namely NRPS-dependent siderophore pathways. Due to the specific functionality of these metal-chelating groups, there have been several studies on how different aryl acids, such as anthranilate, salicylate, and 2,3-dihydroxybenzoate, are enzymatically activated.

**Fig. 11:**
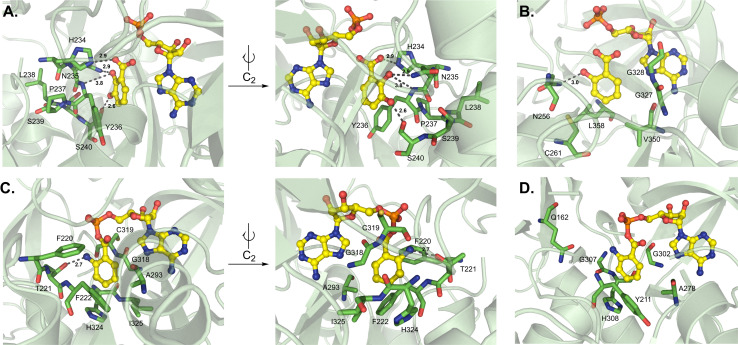
Structures of aryl acid-activating adenylation domains. Residues involved in substrate interactions are shown for (A) 2,3-dihydroxybenzoic acid and AMP in DhbE (PDB 1MD9); (B) salicylate and AMP in CahJ (PDB 5WM2); (C) anthraniloyl-AMP in AuaEII (PDB 4WV3) and (D) anthraniloyl-AMP in PqsA (PDB 5OE3). Active site residues are shown as sticks whereas substrates are highlighted in yellow. Polar contacts are shown as black dashes.

The first aryl acid-activating A domain structure was DhbE from the bacillibactin biosynthetic pathway, which selects for 2,3-dihydroxybenzoic acid (Dhb).^87^ The region that determines Dhb selectivity comprises residues H234-S240 (Fig. 11A). Though the secondary and tertiary structure of DhbE is very similar to GrsA_A, a few of the core A domain motifs were absent. There is no conserved aspartate in DhbE because there is no α-amino group on the substrate to coordinate, which has previously caused some confusion due to the prevalence of D235 in most substrate selectivity codes. However, with the insight provided by structural data, namely the presence of a *cis*Pro241 residue that shifts the peptide backbone, the corrected sequence alignments can now illuminate the importance of N235 and Y236 in Dhb binding (corresponding to D235 and A236 in GrsA_A). N235 forms a hydrogen bond with the 2′-hydroxyl, while S240 hydrogen bonds with the 3′-hydroxyl moiety. Another core motif that confers aryl acid binding is A5, originally annotated as xNxYGPTExx in α-amino acid A domains,^88,89^ but strictly conserved as QQVxFMAEGL here.^87^ Randomizing the residues N235 and V337 in DhbE using yeast cell surface display and probe-based screening showed that the N235Q mutant displayed improved adenylation of 3-hydroxybenzoic acid, as did the A333S and A333T mutants. Interestingly, A333 was not previously thought to be part of the nonribosomal code for aryl acids. A333S and A333T also showed improved binding to 2-aminobutanoic acid (2-Aba), and selection with a 2-Aba probe further led to replacement of V337 with Lys or Arg.^90^ N235 was also necessary for the binding of synthetic aryl-AMP inhibitors, with the equivalent position N242 in BasE forming hydrogen bonds with several compounds in the Dhb binding pocket.^91^

The Dhb-activating A domain EntE from the enterobactin pathway shows remarkable similarity to DhbE.^92,93^ The engineering of EntE to enlarge the substrate binding pocket and accommodate substituted benzoic acids was informed by insights obtained from its crystal structures.^94^ In particular, mutating the conserved D235, which binds to the 2-hydroxyl moiety, to glycine (D235G) widened the entrance of the pocket. The EntE mutant was thus able to bind benzoic acids with bulky substituents at the 2 and 3 positions, namely 3-cyanobenzoic acid and 2-nitrobenzoic acid, in the increased space this mutation provided. It was observed that the 2-nitro group specifically formed hydrogen bonding interactions with the backbone amides of Y236 and A335 (EntE numbering).^94^

In salicylate (Sal) activating A domains, it has been proposed that S240 is replaced by a cysteine to limit the binding of 3-substituted aryl acids. Other residues proposed to mediate this change in selectivity are S239 and V330 (DhbE numbering).^87^ In a mutagenesis study, it was found that residue positions 236, 240 and 339 collectively regulated the specificity of A domains to either Dhb or Sal, with Dhb activation utilizing YSV and Sal activation requiring FCI across the three positions.^95^ The Sal-incorporating A domain CahJ from the cahuitamycin pathway was crystallized in the presence of different substituted monomers.^96^ Much like DhbE and EntE, CahJ maintains residue N256 to interact with the 2-hydroxyl, and as predicted, a C261 to sterically restrict the 3-position, which is joined by L358 (Fig. 11B). The residues surrounding the 4-, 5- and 6-substituted positions around the Sal aromatic ring are C261/L358/V350, V350/G327, and G327/G328, respectively (CahJ numbering). Each of these interaction centers would allow for the binding of Sal analogs with methyl substituents at these positions, though slight rotations of the ring would be required for each, meaning that multiple neighboring methyl groups would not be tolerated.^96^ Indeed, the structures included in this report were bound to benzoate-AMP, Sal-AMP, 5-methyl-Sal-AMP, and 6-methyl-Sal-AMP.

Currently, there are no structures of NRPS A domains that activate unsubstituted anthranilate (Ant), but two CoA ligases bound to Ant-AMP are available as well as one A domain that activates 3-hydroxy-Ant. NpsA is a standalone A domain that initiates tilimycin biosynthesis by activating 3-hydroxy-Ant.^51^ Notable substrate interactions include two hydrogen bonds with the 3-hydroxy moiety mediated by N207 and S271, and a further hydrogen bond between S271 and the 2-amino group (NpsA numbering). Isothermal titration calorimetry (ITC) experiments confirmed that the 3-hydroxyl substituent was more biophysically important than the 2-amino group due to the extra hydrogen bonds it contributes; however, based on *K*_M_ values for the respective substrates, the 2-amino moiety appears to be more important for overall catalytic efficiency.^51^ AuaEII is involved in aurachin biosynthesis,^97^ and PqsA is from *Pseudomonas* quinolone signal (PQS) synthesis.^50^ CoA-ligases do not maintain the same substrate selectivity codes as NRPS A domains, but the structural report of AuaEII listed 19 residues that were critical for binding the various chemical moieties of Ant-AMP (SFTFASEADGIGCTHIDRK).^97^ Only eight of these residues were required for anthranilate interactions (F220, T221, F222, A293, G318, C319, H324 and I325), with F222, A293, H324 and I325 participating directly in aryl interactions (AuaEII numbering) (Fig. 11C). Regarding PqsA, there were four anthranilate-interacting residues noted in the PqsA structure (Y211, A278, G302 and H308, PqsA numbering) (Fig. 11D), as well as several others in its 15 residue binding pocket (QYAGSPDGIGTGHDR).^50^ In PqsA, the α-amino group of Ant was coordinated differently than in AuaEII, utilizing a water molecule to bridge Q162 and the carbonyl of G307. With AuaEII, by contrast, a direct hydrogen bond from T221 (corresponding to G210 in PqsA) was found to bind the α-amino group, indicating that there are two different modes of α-amino recognition in Ant-CoA ligases.^50^

### 2.7 Activation of unique building blocks

**Fig. 12:**
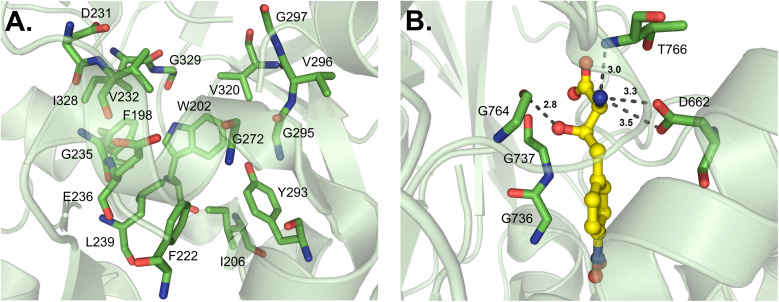
Adenylation domains that activate unique building blocks. (A) Residues lining the binding pocket in SidN (PDB 3ITE). (B) Residues interacting with (2*S*,3*R*)-β-hydroxy-*p*-nitro-homophenylalanine (hnh) in ObiF1 (PDB 6N8E). Active site residues are shown as sticks whereas hnh is highlighted in yellow. Polar contacts are shown as black dashes.

Some A domains activate unique building blocks, and one such pathway is the fungal siderophore enzyme SidN, which generates ferrichromes. Specifically, the third A domain of SidN activates and incorporates *N*^δ^-*cis*-anhydromevalonyl-*N*^δ^-hydroxy-l-ornithine (*cis*-AMHO).^38^ Of note, SidN_A3 is the only fungal adenylation domain that has been crystallized to date. At present, very little is known regarding the apparent divergence of fungal A domain structures and consequent mechanisms of selectivity due to a lack of rigorous studies. It was previously believed that fungal A domains would adhere to bacterial A domain rules, and though there are some broad strokes of similarity, there are also many nuanced differences that have been observed. The crystallized SidN_A3 adopts a very similar fold to other A domains, with A_core_ containing a distorted β-barrel and two β-sheets flanked by α-helices, organized in an overall αβαβα tertiary structure. However, the C-terminal A_sub_ contains three α-helices and one β-sheet, and in total, SidN_A3 lacks three α-helices that are present in GrsA_A while maintaining two additional α-helices not observed in the bacterial enzyme.^38^ The structure of the substrate *cis*-AMHO is large and quite unusual compared to other NRPS building blocks (Fig. 4), and the authors noted that of the 17 identified binding pocket residues in SidN_A3, five were glycine. This was thought to aid in the accommodation of such a large substrate. As the pocket of SidN_A3 is so specific, made up of F198, W202, I206, F222, D231, V232, G235, E236, L239, G272, Y293, G295, V296, G297, V320, I328 and G329 (GrsA_A numbering), it was found to exclusively activate *cis*-AMHO (Fig. 12A). This new 17 amino acid “code” (17AA code B, Table 2) for fungal siderophore synthetases also shared a few residues with previously identified expanded codes.^36,37^ It should be mentioned that because SidN_A3 was crystallized without a substrate, it was in the “open” conformation, and the essential C-terminal lysine residue (K517, GrsA_A numbering) was shifted ∼11 Å away from the binding pocket and not observed to interact with *cis*-AMHO upon docking analysis.^38^

**Table 2:** Comparison of amino acid residue positions of all published, non-substrate specific A domain codes. All numbering is based on GrsA_A (aka PheA, 1AMU)

10AA code^18^	9AA code^34^	13AA code^36^	17AA code A^37^	17AA code B^38^	15AA code^39^	18AA code^109^
				210		
				214		
				218		
		226		226		
		229	229			
			230			
					234	234
235	235	235	235	235	235	235
236	236	236	236	236	236	236
239	239	239	239	239	239	239
			240	240		
			243	243		
		276				
278	278	278	278	278	278	278
			280			
				297		
299	299	299	299	299	299	299
				300		
301	301	301	301	301	301	301
					302	302
			320			
322	322	322	322	322	322	322
					323	323
					324	324
					325	325
			326			326
						329
330	330	330	330	330	330	330
331		331	331	331	331	331
517	517	517	517		517	517
						519

ObiF1 is a unique NRPS module that activates (2*S*,3*R*)-β-hydroxy-*p*-nitro-homophenylalanine (hnhF) in obafluorin biosynthesis. Similar to Thr1, the substrate β-hydroxy is coordinated by main chain atoms instead of residue side chains, namely G736, G737 and G764 (ObiF1 numbering) (Fig. 12B). The orientation of G764 specifically may be influenced by I765, conserved in Thr1, to allow for a hydrogen bond to form between the G764 carbonyl and the β-hydroxyl group of hnhF. The partial substrate selectivity code of ObiF1 is 
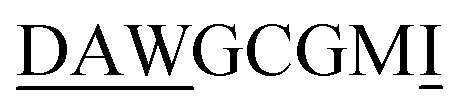
, which shares four positions with the GrsA_A code 
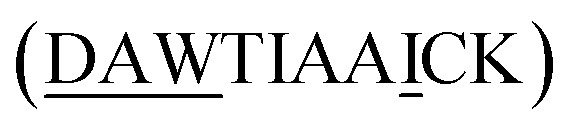
, but the differences provide a hydrophobic pocket for the *p*-nitro-phenyl group to avoid the otherwise polar, charged contacts along the peptide backbone. The α-amino group of hnhF participates in a hydrogen bond with the amide proton of T766 and a salt bridge with D662.^98^

**Fig. 13:**
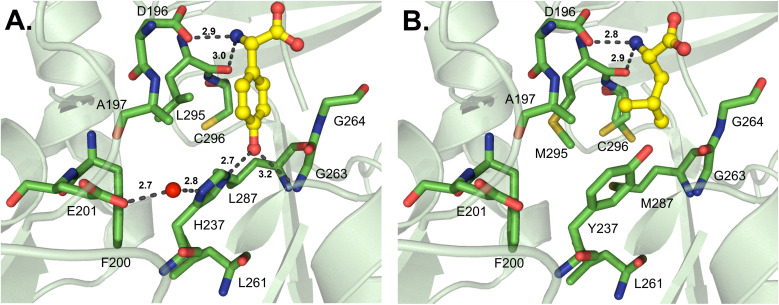
Structures of teicoplanin and teicoplanin-like adenylation domains. Residues involved in substrate interactions are shown for (A) l-Hpg in Tcp9_A1_core-tei_ (PDB 8GIC); and (B) l-leucine in the mutated Tcp9_A1 variant Tcp9A1_core-ANC3_ (PDB 8GKM). Active site residues are shown as sticks whereas substrates are highlighted in yellow. Polar contacts are shown as black dashes and the water molecule coordinating residue H237 and E201 in panel A is designated as a red sphere.

While this article was under review, new work from the Cryle and Ziemert groups was published describing several A domain crystal structures, particularly those from glycopeptide antibiotic pathways.^99^ Tcp9_A1, which activates both l- and d-4-hydroxyphenylglycine (4-Hpg) in the first step of teicoplanin biosynthesis, was crystallized bound to l-4-Hpg. The aromatic ring of the substrate interacts with the side chain of L295, and the hydroxyl group at position 4 forms a hydrogen bond to H237 (Tcp9_A1 numbering) (Fig. 13A). Of note, the side chain of H237 is oriented by hydrophobic interactions with L261 and L287, and a water molecule bridges a hydrogen bond between the imidazole group and a conserved E201 residue. The authors made a handful of key mutations in Tcp9_A1 to emulate the binding pocket residues of ancestral enzymes that had different selectivity profiles. Vancomycin-type glycopeptide antibiotics replace d-4-Hpg with d-leucine, while those of the pekiskomycin-type activate d-Ala. By making the mutations H237Y and L295V, 4-Hpg binding was eliminated and the pocket was distorted to accommodate non-planar proteinogenic substrates, however enantiomeric selectivity and catalytic efficiency were reduced. The further mutated enzyme H237Y/L287M/L295M has improved van der Waals contacts with l-Leu, providing stereoselective activation and marginally improved catalysis (Fig. 13B). The authors were also able to crystallize an ancestral d-Ala-activating A domain, and though there was no substrate bound, they noted that the binding pocket was significantly smaller.^99^

## 3 Development of various specificity “codes”

Several studies have identified specific positions of A domain residues that can be used in primary amino acid sequence alignments to predict substrate selectivity for newly discovered or uncharacterized A domains. There is some debate over the significance of residues that dictate substrate recognition by discrete chemical interactions (the “first shell”) as opposed to residues that perform a more general structural role in the binding pocket (the “second shell”). The first report canonized the 10 amino acid code (10AA code or Stachelhaus code) of GrsA_A as a fingerprint for A domain substrate selectivity in bacteria,^18^ which was quickly followed by another group corroborating nine of the ten residues.^34^ The 10AA code has historically provided the best predictions for canonical bacterial A domains that activate l-α-amino acids. These 10 residues can also be identified in fungal A domains,^44^ though with variable predictive success. Despite its incorporation into many *in silico* prediction tools, it became clear that the 10AA code was not accurate in every case, leading to reports of expanded codes, many of which were determined from different A domain structures. A comparison of all codes that map onto GrsA_A numbering is shown in Table 2. Several of these expanded codes have been proposed based specifically on fungal A domain sequences, which helps to fill a significant gap in our current predictive abilities.^36–38^ Studies on fungal systems has either focused solely on specific types of NRPSs, like siderophore synthetases^36,37^ or anthranilate-activating A domains,^100^ or have provided a broader understanding of the general differences among fungal adenylating enzymes.^101^ Schwecke *et al.* first described siderophore synthetases in fungi using comparative modeling with the MODELLER program.^36,102,103^ They observed that the 10AA code was still present, but three additional residues were found to be essential for substrate binding, generating a 13AA code.^36^ Bushley and co-workers reported a 17AA code (17AA code A, Table 2) based on evolutionary relationships of fungal NRPSs and not structural analysis.^37^ Upon crystallization of the first fungal A domain, which activated a bulky hydroxamate substrate, a second 17AA code was defined (17AA code B, Table 2), as discussed in Section 3.7.^38^ Despite multiple reports focused on fungal A domains, all of these studies have looked at siderophore pathways, which might not directly translate to other NRPS A domains.

It should be noted that a 34 residue code exists based on an 8 Å distance around the A domain active site, measured by Rausch *et al.*, but no residue positions were reported for comparison to existing bacterial codes.^35^ The 15AA code reported by Khurana and co-workers was determined by measuring a 6 Å distance around a docked substrate in homology models of members of the acyl:CoA synthetase superfamily.^39^ Several computational and directed evolution experiments have also confirmed the importance of some residue positions, specifically sites 278 and 301 (either one or both).^104–107^ A phylogenetic study of all classes of fungal adenylating enzymes recently confirmed the importance of the first positions of the code, namely residues 234, 235, 236, and 239, but noticed that the fungal A domain code diverges significantly from well-studied bacterial examples.^101^ The question of a fungal A domain selectivity code has been addressed by several groups as described here, and we have contributed an 18AA code for fungal A domains and activation of noncanonical building blocks. In brief, homology models generated by AlphaFold^108^ were used to determine all residues within a radius of 5 Å of the active site, and this putative code was then biochemically verified by mutagenesis of a characterized fungal A domain.^109^ It appears that structural data is indeed a superior predictor of substrate preference than primary sequence alignments alone. Nevertheless, strong selectivity predictions still require mutagenesis and biochemical interrogation to verify key positions due to the risk of deforming the binding pocket.

## 4 Biochemical characterization of adenylation domains

**Fig. 14:**
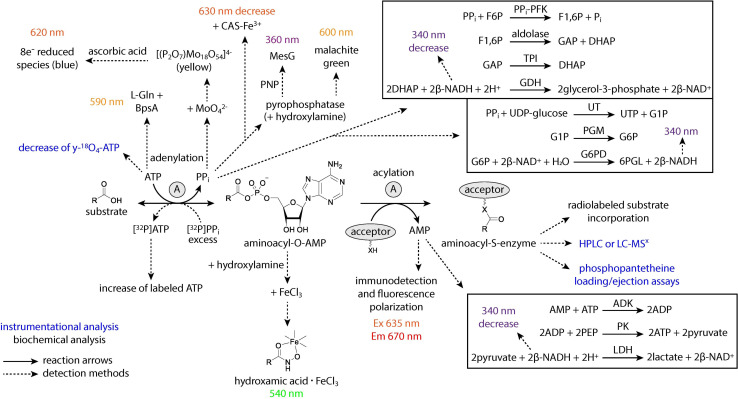
*In vitro* methods for interrogating adenylation domain substrate specificity. Solid arrows represent the adenylation reaction, and dashed arrows represent the various detection methods branching from it. Blue text indicates methods that require instrumentational analysis, while black text indicates biochemical methods. Colorimetric and fluorometric assays are color-coded based on the wavelength of light they measure. ATP = Adenosine triphosphate, ADP = adenosine diphosphate, AMP = adenosine monophosphate, PP_i_ = inorganic pyrophosphate, P_i_ = inorganic phosphate, CAS = Chrome Azurol S, NADH = nicotinamide adenine dinucleotide reduced, NAD^+^ = nicotinamide adenine dinucleotide, l-Gln = l-glutamine, F6P = fructose-6-phosphate, PP_i_-PFK = PP_i_-dependent phosphofructokinase, F1,6P = fructose-1,6-bisphosphate, GAP = glyceraldehyde-3-phosphate, DHAP = dihydroxyacetone phosphate, TPI = triose phosphate isomerase, GDH = glycerol-3-phosphate dehydrogenase, UDP = uridine diphosphate, UT = glucose-1-phosphate uridylyltransferase, UTP = uridine triphosphate, G1P = glucose-1-phosphate, PGM = phosphoglucomutase, G6P = glucose-6-phosphate, G6PD = glucose-6-phosphate dehydrogenase, 6PGL = 6-phosphono-d-glucono-1,5-lactone, ADK = adenosine kinase, PEP = phosphoenolpyruvate, PK = pyruvate kinase, LDH = lactate dehydrogenase, HPLC = high performance liquid chromatography, LC-MS^x^ = liquid chromatography tandem mass spectrometry.

Regardless of structural data supporting substrate activation and key interactions in the binding pocket, the biochemical role of A domains must still be confirmed. There are a wide variety of assays available to gain a better understanding of A domain function. Some assays measure the adenylation half-reaction, while others measure the ability of an activated substrate to be loaded onto an acceptor molecule or carrier protein. These assays also vary in their detection methods, and it is our hope that by compiling a list, other researchers will be able to select whichever method is best for them depending on the availability and access to different resources. The different methods are summarized in Fig. 14.

Some general patterns emerge from the various techniques and reports on adenylation activity. First, many of the early methods measured the endpoint of a reaction and were not conducive to continuous measurements for kinetic analysis of A domains, instead requiring multiple separate reactions for each time point. We have delineated which of the methods below can be used in a continuous manner to reduce the time-consuming and resource-draining setup. Second, the addition of an acceptor molecule (either hydroxylamine or a downstream carrier protein) to the adenylation reaction, even when only the first half-reaction is being measured, is not only more physiologically relevant but often improves the reaction kinetics and encourages rection completion. However, the use of an exogenous acceptor can distort results, especially when investigating nonstandard substrates or mutant enzymes that may have increased spontaneous release of their intermediates. Some assays found in primary literature may vary from the general procedures listed below. Our goal is to report first and foremost on the detection method and juxtapose it to others, leaving readers to determine which option best applies to their system.

The ability to measure the progress of a reaction over time is necessary in general biochemistry. Some reactions consist of a single step and generate few if any intermediates, but the adenylation reaction is a two-step process with multiple reactants, products, and co-substrates. While this makes measurement of adenylation activity possible at many points along the reaction pathway (Fig. 14), it is important to understand each minor event and how different assays account for the dynamic interactions between multiple substrates, side products and other enzymes over the course of the reaction. Specifically, adenylation begins when an A domain binds both ATP and its cognate substrate in adjacent active site pockets. Hydrolysis of ATP produces PP_i_, which exits the active site, and an AMP-tethered intermediate. The adenylated substrate remains in the binding pocket until a nucleophilic attack catalyzed by the free thiol on the Ppant arm of a neighboring T domain, at which time the high-energy acyl-AMP is transferred, releasing AMP from the active site. There are many proposed conformational rearrangements of the A_core_, A_sub_ and T domains that must take place for each step of the reaction cycle to proceed, and many of these conformations have been captured in the structural studies discussed above (Fig. 2). The order of various techniques presented in the following section begins with direct methods of substrate detection and ends with indirect methods, moving in reaction chronology (Fig. 14).

Some strategies that will not be discussed in detail here include the body of work pioneered by the Ishikawa and Kakeya labs in the realm of chemical proteomics and activity-based protein profiling.^110,111^ Building on work that designed and synthesized clickable probes^82^ and inhibitors of A domains,^55–57^ Ishikawa and coworkers have established methods that can detect and interrogate endogenous NRPSs using ELISA^112–114^ and *in vivo* techniques.^115,116^ This has provided valuable insight into chemical moieties required for binding and remodeling of active site architecture, while avoiding the labor-intensive and problematic enzyme purifications that many other workflows require. These contributions, while significant to the field, are often untargeted or exploratory in nature and require the generation of synthetic probes for each tested substrate. Thus, the workflow is not amenable to the comprehensive study of substrate selectivity in previously identified A domains of interest. The methods we have chosen to focus on are broadly applicable to any adenylation enzyme activating any acyl-containing substrate and can be performed without extensive technical knowledge. While many of the assays discussed in this section have been summarized elsewhere,^15,117^ this review, to the best of our knowledge, represents the most comprehensive compilation of biochemical methods for analyzing the adenylation reaction. As several of the methods discussed below detect a chemical species after it diffuses out of the active site, caution should be exercised when interpreting results. The leakage of any reaction product (acyl-AMP, PP_i_ or AMP) from the binding site can vary widely from enzyme to enzyme and can account for a significant portion of observed activity depending on the detection method being used.^118,119^

### 4.1 Radiolabeling and colorimetric assays

The most direct assay to measure substrate adenylation is the colorimetric hydroxylamine-trapping iron complex method.^120,121^ Hydroxylamine is used as an acceptor molecule, displacing AMP to form a hydroxamic acid with the acyl substrate that can be detected spectroscopically upon addition of a solution containing Fe^3+^. This assay is not dependent on PP_i_ release from the A domain, but drawbacks include discontinuity, low sensitivity, instability of the complex, and the observation that not all hydroxamate–iron complexes are detectable spectroscopically. Another direct detection method is the radiolabeled formation of a PCP-substrate species. This reaction requires an activated PCP domain and the addition of a radiolabeled substrate (usually ^3^H, ^14^C, or ^35^S), which can then be measured after precipitation of the protein using trichloroacetic acid (TCA).^122,123^ Due to the necessity of separating the enzyme from the remaining aqueous labeled substrate, this assay is discontinuous, and its utilization of radioactive materials makes it hazardous and inconvenient.

Indirectly measuring adenylation activity can be just as accurate as direct detection depending on the specific method providing the readout. Recently, the Ackerley group described a colorimetric method that quantifies residual ATP present in the reaction mixture after adenylation.^124^ The protein BpsA is a single module NRPS consisting of an A-Ox-T-TE domain organization, and it is known to catalyze the formation of the blue compound indigoidine from two molecules of l-glutamine and two molecules of ATP. By adding BpsA and excess l-Glu to an A domain reaction after incubation, the consumption of ATP is stoichiometrically measured as the inverse to the amount of indigoidine formed. This assay provides a more sensitive color change than related malachite green methods listed below, but it does not allow for continuous measurement.

Possibly the largest number of adenylation assays involves indirectly measuring activity *via* the release of PP_i_ from the enzyme. There is some discussion surrounding the applicability of PP_i_ release assays to various A domains, as in some cases, the pyrophosphate remains tightly bound to the enzyme active site. The first of these is a mainstay in the NRPS field, having been used most frequently for analysis of diverse systems: the ATP-[^32^P]PP_i_ exchange assay.^125,126^ Dependent on the reverse hydrolysis of ATP in the first half-reaction, excess radiolabeled PP_i_ is added to the reaction, and the resulting ^32^P-ATP is adsorbed on activated charcoal before washing and liquid scintillation counting. In early days, this assay required large volumes of hazardous and expensive radioactive materials, and it was time-consuming and technically demanding. More recently, a high-throughput, 96-well plate optimized procedure has become more feasible.^126^ This PP_i_ exchange assay is discontinuous, but kinetic constants derived from it are a good approximation of physiologically relevant reaction rates. Other colorimetric, discontinuous strategies for measuring PP_i_ release utilize the malachite green P_i_ detection assay or direct precipitation of PP_i_ as an 18-molybdopyrophosphate anion. The malachite green method involves the addition of a pyrophosphatase enzyme to the reaction mixture, which rapidly converts any released PP_i_ to P_i_. Established phosphate detection assays can then be used, with the addition of molybdate and malachite green providing a color change that can be read at 600 nm.^127,128^ This method is easy to use and the reagents are readily accessible, considering that commercial phosphate detection kits are common. Molybdate-PP_i_ precipitation generates a [(P_2_O_7_)Mo_18_O_54_]^4−^ species that can then be further reduced by ascorbic acid to give a more distinguishable color change.^129–131^ However, turnover rates of A domains in PP_i_ release assays are thought to be much slower than those from PP_i_ exchange assays due to the lack of an acceptor nucleophile, leading to dependence on PP_i_ leakage from the active site for accurate measurement. Additionally, phosphate is a common contaminant, leading to high background signals. By contrast, a continuous colorimetric readout exists in the form of the MesG assay. Based on older reports of phosphate detection assays and the color-developing conversion of 7-methylthioguanosine (MesG) to 7-methylthioguanine by the enzyme PNP,^132–136^ the coupling of this enzyme activity to both the adenylation reaction and a pyrophosphatase has been used extensively. More recently, the Aldrich group has adapted this assay specifically for A domains by addition of a hydroxylamine acceptor molecule.^118,119^ This hydroxylamine-MesG workflow represents a convenient alternative to radioactive PP_i_ exchange assays with very few drawbacks. The Townsend group also reported recently an improved but discontinuous iron-based colorimetric method employing Chrome Azurol S (CAS),^117^ a reagent that chelates iron and has historically been used to detect siderophores. This assay uses the same setup as the original hydroxylamine-trapping iron complex method, but the addition of a CAS-Fe^3+^ mixture provides a more sensitive and rapid color change. The authors specifically noted that it was PP_i_ that acted as the predominant iron-sequestering moiety and not the substrate hydroxamate, a property not exhibited by orthophosphate alone. It was recommended that a pyrophosphatase be added to iron-hydroxamate workflows to remove metal binding competition by PP_i_ and improve sensitivity even without the addition of CAS-Fe^3+^.^117^

Exploiting reversible reactions from primary metabolic pathways is a common strategy in the development of continuous, NAD^+^/NADH-coupled assays. There are two such examples that fall in the category of adenylation PP_i_ release, where pyrophosphate formation is connected to NADH generation or consumption *via* the activity of one or more enzymes. Some coupled assays are able to amplify the detectable adenylation activity by using enzymes that stoichiometrically increase NADH output. The first example couples the activity of three enzymes to the A domain reaction: glucose-1-phosphate uridylyltransferase (UT), phosphoglucomutase (PGM), and glucose-6-phosphate dehydrogenase (G6PD).^104,137,138^ The second example involves four coupled enzyme reactions, the most of any reported here: PP_i_-dependent phosphofructokinase (PP_i_-PFK), aldolase, triose phosphate isomerase (TPI), and glycerol-3-phosphate dehydrogenase (GDH).^139,140^ It should be noted that the first of these methods measures an increase in NADH, and the second measures a 2-fold decrease. In general, coupled assays are more complicated because they rely on the additional enzymes reacting fast enough that, especially for continuous measurements, their influence on the overall reaction rate (from PP_i_ release to detectable NADH generation or consumption) is almost negligible.

Finally, two *in vitro* methods have been developed for the quantification of AMP released from an A domain in the second half-reaction. A continuous colorimetric AMP release assay is achieved by coupling the adenylation reaction to NADH consumption. The decrease in NADH is measured with the addition of adenosine kinase (ADK), pyruvate kinase (PK) and lactate dehydrogenase (LDH).^141,142^ Unfortunately, this assay suffers from similar issues as other NADH-coupled assays, namely high background activity and reliance on three additional enzymes. To avoid these issues, a novel discontinuous AMP release assay was developed by Staeben *et al.* that uses immunodetection of AMP and far red fluorescence polarization for quantification.^143^ This assay, though not widely used in the NRPS community, displays good sensitivity and avoids having to couple adenylation activity to one or more additional enzymes. The assay reagents were shown to be stable for 24 hours at room temperature, but the cost and specific handling of antibodies, as well as the instrumentation required, make this protocol somewhat less accessible.

### 4.2 Mass spectrometry-based methods

Early mass spectrometry (MS)-based methods to interrogate NRPS pathways originated from related proteomics-based approaches, and therefore they often involve interrogation of the carrier protein or T domain of a pathway. T domains are small in size (<15 kDa), lending to their facile detection and distinguishable mass shifts when bound to substrates and/or cofactors. There are two types of MS approaches that can be used: top-down or bottom-up. Top-down MS analyzes intact proteins, often stand-alone T domains, though larger constructs have also been successfully detected. Many top-down MS workflows use ion trap instruments with lower resolution or Fourier-transform mass spectrometry (FTMS) with much higher resolution. FTMS is ideal for detecting mass increases as low as 1 Da, but these instruments tend to be custom builds and/or very expensive. In bottom-up MS workflows, *in vitro* reactions with NRPSs are performed, followed by digestion, either enzymatically with trypsin or chemically with cyanogen bromide. The resulting peptides are separated by reverse phase liquid chromatography (LC) before being individually analyzed by FTMS, often with tandem MS (MS^2^). Most of these techniques have been discussed elsewhere in more detail,^144–146^ but we provide a summary and comparison below. It should be noted that MS in general is a destructive detection method, meaning that samples are measured at an end point and data often cannot be collected in a continuous manner.

Several MS-based methods of A domain substrate activation directly detect the PCP-bound thioester moiety.^145^ The first of these is the Ppant-loading assay, which is a bottom-up approach using either FTMS or matrix-assisted laser desorption ionization time-of-flight (MALDI-ToF) mass spectrometry, measuring the mass shift observed when a Ppant-activated PCP is incubated with a pool of available substrates and then digested for analysis.^147–149^ This allows for an unbiased screening of A domain activity, but the composition of the substrate pool must be known in order to determine the identity of the activated monomer by mass shift alone. A top-down iteration of this method gave rise to the Ppant-ejection assay using either tandem MS capabilities (MS^2^ or MS^3^) or collision induced dissociation (CID) to fragment an intact substrate-tethered PCP. Either FTMS or ion trap MS can be used in Ppant-ejection assays, and the detected mass corresponds to the substrate covalently bound to either the intact Ppant arm or a shortened, rearranged pantetheine prosthetic group.^150,151^ The move to a top-down workflow circumvents the need to proteolyze samples before analysis, and the validation of the technique on lower resolution instruments such as ion trap MS improves accessibility.

Another possibility with instrumentational analysis of A domains is the perhaps obvious ability to detect adenylated intermediates directly. This relies on the leakiness of A domains, which might allow the substrate-AMP complex to dissociate from the active site after activation, which is not the case for all enzymes. Indeed, in practice, not all substrate-AMP intermediates can be detected in reaction solutions. However, in a reasonable number of cases, high performance liquid chromatography (HPLC) or liquid chromatography coupled to mass spectrometry (LC-MS) can be used to detect AMP-bound substrates directly.^152–154^ To its credit, this method does allow for kinetic profiling of adenylation reactions. By invoking tandem MS and a similar workflow to that of the Ppant ejection assay, the multiplexed hydroxamate assay (HAMA) was developed. In this workflow, A domains are incubated with a defined pool of substrates before addition of a hydroxamate acceptor, which converts the substrate-AMP to a hydroxamic acid conjugate detectable by LC-MS.^2^ The initial report on HAMA highlighted the importance of analyzing A domains exposed to a substrate pool, which more accurately recapitulates the competition conditions experienced *in vivo* and therefore deduces more precise kinetic constants such as *k*_cat_/*K*_M._^155^ Unfortunately, hydroxamate quantification by LC-MS^2^ requires the generation of synthetic standards for calibration and optimization, which is not chemically possible for all proteinogenic and nonproteinogenic building blocks. Further, the study noted that detection of some hydroxamates was complicated by either isobaric, coeluting compounds present in the assay mixture or deuterium labelling required to differentiate between enantiomeric pairs of certain amino acids.

A final method of MS-based A domain interrogation has been reported using nonradioactive isotopic labeling in a manner reminiscent of PP_i_ exchange assays. Phelan *et al.* describe the indirect measurement of PP_i_ exchange by initiating A domain assays with γ-^18^O_4_-ATP, which will release its labeled γ-phosphate as PP_i_ in the forward adenylation reaction. As with the radiolabeled ^32^P[PP_i_] assay, this technique relies on the reverse reaction, incorporating unlabeled PP_i_ to form γ-^16^O_4_-ATP. The rate of γ-^16^O_4_-ATP formation and γ-^18^O_4_-ATP consumption can therefore be observed by mass shift, and the integrated peak ratio of γ-^16^O_4_-ATP to all ATP species present allows for quantification of enzyme activity.^156^ This assay can be performed on either a MALDI-ToF instrument or electrospray ionization (ESI) LC-MS instruments, but it is a discontinuous method to probe reactions.

## 5 Bioinformatic analysis of adenylation domains

As it has become more affordable to sequence the genomes of new organisms, and more streamlined to genome mine for new biosynthetic gene clusters, several *in silico* tools have been developed over the last few decades for the bioinformatic prediction of A domain substrate selectivity in NRPS pathways. It should be noted that the best predictor of substrate selectivity, as discussed above, is structural data. However, after the 10AA code was established, primary amino acid sequence alignments of uncharacterized proteins with domains of known structure or selectivity became the most common method of comparison. Sequence alignments are quick, cheap, and easy to generate, but they are not always the most accurate method for substrate prediction. Caution needs to be taken as the 10AA code does not accurately apply to all enzymes and organisms. Further, when interrogating novel pathways preforming unique chemistry, there are often no homologous examples to use as a reference, making predictions tenuous at best. Finally, not all *in silico* tools or platforms use the same method of substrate prediction, and devastatingly, many of them have not been maintained over the years. It is our hope that the natural product research community will endeavor to uphold a wide variety of bioinformatic platforms, which will allow for more accurate predictions in the future, and the subsequent isolation of new bioactive molecules. Two somewhat recent comprehensive reviews of various platforms^157^ and alignment-free methods^158^ are available, and we present in the sections below an updated and abbreviated NRPS-focused comparison.

### 5.1 Currently available tools

By far, the most popular and well-rounded bioinformatics platform is antiSMASH, of which substrate prediction is just one component.^159^ antiSMASH and its accessory programs (MIBiG,^160,161^ BiG-SCAPE, and CORASON^162^) are the Swiss Army pocketknife of natural products bioinformatic tools, being widely used in the field and regularly maintained and updated for relevancy. The latest update to antiSMASH has incorporated an enhanced phylogenetics-based algorithm ensemble for improved NRPS A domain prediction.^163^ There is also a Hidden Markov Model (HMM)-based approach that has been incorporated, which attempts to predict protein–protein docking regions between modules in addition to substrate specificity.^164^ The greatest strength of antiSMASH is its utilitarian ability to identify several types of biosynthetic gene clusters across a wide swath of organisms. However, this means that concise predictions for a specific type of enzyme may not always be successful, especially when similarity to known enzymes is low. There is a noticeable drop-off in prediction accuracy with antiSMASH in cases where there are fewer characterized examples, which will improve with the biochemical characterization of new clusters.

There are many other tools that have emerged over the years to aid researchers in natural product drug discovery and dereplication of known compounds. As polyketides and nonribosomal peptides are two of the largest classes of natural products, and their respective enzymatic machinery generally follow a colinear, assembly line-like organization of active site domains, the prediction of PKS and NRPS substrates was of early intrigue. Prieto and co-workers developed the NRPS substrate predictor (NRPSsp), which uses an HMM database for its predictions.^165,166^ The PRISM 4 platform is a comprehensive tool for the HMM-based prediction of antibiotic structures from bacterial genomes regardless of their natural product class.^167^ Two currently active tools released in 2020 have combined random forest models and HMMs for improved predictions: AdenylPred uses machine learning to predict general adenylation preferences in all class I adenylate-forming enzymes from bacteria, fungi and plants,^168^ and SeMPI 2.0 predicts chemical structures including an emphasis on post-assembly line modifications.^169^ The latter also screens scaffolds against public gene cluster databases in an attempt to connect genomic data to compounds and thus estimate novelty. Most recently, the Mohimani group developed AdenPredictor, which uses unsupervised machine learning and the extra trees model paired with one-hot encoding features in its predictions. The authors also benchmarked existing popular *in silico* tools, which revealed that for substrate specificity inquiries, the most reliable models are extra trees and logistic regressions.^170^ To the best of our knowledge, the web tools listed above are the only currently active substrate prediction methods, as many more have become unavailable in recent years.

### 5.2 Discontinued and obsolete platforms

Bachmann and Ravel developed a PKS/NRPS predictive BLAST server in 2009 that currently does not function. The server detected catalytic domains in PKSs and NRPSs, comparing signature motifs of A domains with those of characterized proteins and known substrates.^171^ The same year, the alignment-based tool NP.searcher was launched, which exclusively used BLAST to identify signature motifs and predict nonribosomal peptide and polyketide structures from microbial genomes.^172^ A novel *in silico* platform that has only recently disappeared is NRPSpredictor2, which used transductive support vector machines (TSVMs) to classify A domains according to their substrate preferences as a foundation for predictions.^35,173^ Another HMM-based approach was the NRPS/PKS substrate predictor tool, which may have become obsolete upon addition of HMM predictions to larger platforms like antiSMASH.^174^ One of the most accurate tools for substrate prediction was the LSI function predictor. This A domain-based algorithm used Latent Semantic Indexing (LSI) on FASTA input files and was a staple of early genome mining efforts.^175^ And finally, a relatively recent webserver developed in 2016, SEQL-NRPS has already been discontinued, but it used Sequence Learner (SEQL) as a discriminative classification method for A domains.^176^ Many of these web tools are either inaccessible from the provided URL, or else the only response that is generated is an error message. It is unclear thus far the reasons for the lack of maintenance of various *in silico* algorithms and web tools, but contributing factors may include funding, tedious manual curation to avoid obsolescence, and a deficiency in enthusiasm or assistance from the community. It should be noted that in recent years, the development of machine learning algorithms for genome mining has become more complex. Many of these methods, though quite accurate, are more computationally demanding and are not hosted through an online server, requiring command-line execution and making them less accessible to researchers lacking a background in computer science. The adaptation of an *in silico* platform into a user interface is no small feat, but it would be beneficial if modern and more advanced methods of substrate prediction could be hosted through web-based applications, at least in addition to their code being publicly available.

## 6 Conclusions

A domains embedded in NRPS pathways show enormous flexibility in terms of the diverse building blocks they are able to activate and incorporate into nonribosomally-derived peptides. Significant work has already illuminated a vast array of A domain structures, both stand-alone and as part of a module, leading to an enhanced understanding of the molecular mechanisms required for activation of different classes of substrates. This has allowed for the determination of various substrate selectivity codes, which can be biochemically verified using a plethora of *in vitro* methods, and whose detection can be either chemical or instrumentational. Further, the continued curation of *in silico* algorithms and web tools provide a means to interrogate A domains and their predicted substrate scope using only genomic data, which continues to push natural product drug discovery towards a very bright future.

## 7 Conflicts of interest

There are no conflicts to declare.
